# Women’s values and preferences on low-molecular-weight heparin and pregnancy: a mixed-methods systematic review

**DOI:** 10.1186/s12884-022-05042-x

**Published:** 2022-10-05

**Authors:** Montserrat León-García, Brittany Humphries, Andrea Maraboto, Montserrat Rabassa, Kasey R. Boehmer, Lilisbeth Perestelo-Perez, Feng Xie, Irene Pelayo, Mark Eckman, Shannon Bates, Anna Selva, Pablo Alonso-Coello

**Affiliations:** 1 Biomedical Research Institute Sant Pau (IIB Sant Pau), Barcelona, Spain; 2 Department of Pediatrics, Obstetrics, Gynaecology and Preventive Medicine, Universidad Autónoma de Barcelona, Barcelona, Spain; 3 Knowledge and Evaluation Research Unit, Department of Medicine, Mayo Clinic, Rochester, MN USA; 4 Cytel Inc, Toronto, ON Canada; 5 Department of Health Research Methods, Evidence and Impact, McMaster University, Hamilton, ON Canada; 6 Division of Health Care Delivery Research, Mayo Clinic, Rochester, MN USA; 7 Evaluation Unit (SESCS), Canary Islands Health Service (SCS), Tenerife, Spain; 8 Research Network On Health Services in Chronic Diseases (REDISSEC), Tenerife, Spain; 9 Network for Research On Chronicity, Primary Care, and Health Promotion (RICAPPS), Tenerife, Spain; 10 Centre for Health Economics and Policy Analysis, McMaster University, Hamilton, ON Canada; 11 Department of Obstetrics and Gynecology, Ramón y Cajal Hospital, Madrid, Spain; 12 Faculty of Medicine, Alcalá de Henares University, Madrid, Spain; 13 Division of General Internal Medicine and Center for Clinical Effectiveness, University of Cincinnati School of Medicine, Cincinnati, OH USA; 14 Department of Medicine, McMaster University, Hamilton, ON Canada; 15 Clinical Epidemiology and Cancer Screening, Corporació Sanitaria Parc Taulí, Sabadell, Barcelona, Spain; 16 CIBER of Epidemiology and Public Health, CIBERESP, Madrid, Spain

**Keywords:** Venous thromboembolism, Low-molecular-weight-heparin, Pregnancy, Values and preferences

## Abstract

**Background:**

Venous thromboembolism (VTE) in pregnancy is an important cause of maternal morbidity and mortality. Low-molecular-weight heparin (LMWH) is the cornerstone of prophylaxis and treatment of thrombotic events during pregnancy. LMWH has fewer adverse effects than other anticoagulants, does not cross the placenta, and is safe for the fetus. However, the use of LMWH during pregnancy is sensitive to womens’ underlying preferences. The objective of this review is to systematically assess women’s values and preferences research evidence on this topic.

**Methods:**

We searched four electronic databases from inception to March 2022, and included studies examining values and preferences of using LMWH among pregnant women at risk of VTE. We followed a convergent integrated mixed-methods design to compare and contrast quantitative outcomes (utility and non-utility measures) and qualitative findings. We assessed the certainty of the values and preferences evidence with the GRADE approach for quantitative findings, and with GRADE-CERqual for qualitative evidence. Results were presented in a conjoint display.

**Results:**

We screened 3,393 references and identified seven eligible studies. The mixed methods analysis resulted in four themes. Datasets confirmed each other in that: 1) the majority of women consider that benefits of treatment outweigh the inconveniences of daily injections; and 2) main concerns around medication are safety and injections administration. Quantitative outcomes expanded on the qualitative findings in that: 3) participants who perceived a higher risk of VTE were more willing to take LMWH. Finally, we found a discrepancy between the datasets around: 4) the amount of information preferred to make the decision; however, qualitative data expanded to clarify that women prefer making informed decisions and receive support from their clinician in their decision-making process.

**Conclusions:**

We are moderately confident that in the context of pregnancy, using LMWH is preferred by women given its net beneficial balance. Integrating data from different sources of evidence, and representing them in a jointly manner helps to identify patient’s values and preferences. Our results may inform clinical practice guidelines and support shared decision-making process in the clinical encounter for the management of VTE in the context of pregnancy.

**Supplementary Information:**

The online version contains supplementary material available at 10.1186/s12884-022-05042-x.

## Background

Venous thromboembolism (VTE) in pregnancy is an important cause of maternal morbidity and mortality in developed countries [1], responsible for approximately 1.5 to 2% of maternal deaths during pregnancy and the postpartum period [2, 3]. The normal hypercoagulable state during pregnancy increases the risk of developing VTE by 5- to tenfold compared with non-pregnant women [2, 3]. Other medical conditions in pregnancy, such as inherited or acquired risk factors for thrombosis (thrombophilia), can also increase risk of VTE and poor pregnancy outcomes, including placental abruption, preeclampsia, fetal growth restriction, stillbirth, and recurrent miscarriage [4]. Low-molecular-weight heparin (LMWH) is the cornerstone of prophylaxis and treatment of thrombotic events during pregnancy and the postpartum period [5]. LMWH has fewer adverse effects than other anticoagulants, does not cross the placenta, and is safe for the fetus [6, 7]. However, the use of LMWH during pregnancy is challenging, as it is expensive, uncomfortable to administer, may be associated with an increased risk of major obstetrical bleeding, and may jeopardize the use epidural analgesia [7–10].

Like many other decisions in health care, the prevention of VTE during pregnancy does not have a single best option. Many factors influence the decision-making process, and, therefore, it is considered a preference-sensitive decision [11]. This is probably one of the reasons why in a recent critical appraisal of guidelines for the prevention and treatment of pregnancy-associated VTE, recommendations were inconsistent [5]. Similarly, one “strongly recommended for use in practice” guideline included in the critical appraisal, the American Society of Hematology 2018, concluded that healthcare professionals should make decisions through a shared decision-making (SDM) process, incorporating patients’ values and preferences [12].

Two previous reviews have addressed the topic of values and preferences in thrombosis [11, 13]. These reviews show that patients’ values and preferences appear to be highly variable. However, to date, there is not a specific review addressing women’s values and preferences for antithrombotic therapy during pregnancy. Furthermore, one of these reviews [11] only included quantitative measures, while the other [13] collected information from both quantitative and qualitative measures, but synthesized the information independently and did not integrate findings. Good Reporting of A Mixed Methods Study (GRAMMS) criteria have highlighted this limitation, in which judgements about integration could rarely be made due to the lack of integration of data and findings [14]. Therefore, this review contributes to the field of mixed methods research by using an integrative- convergent design [15] that are optimal to conduct the study of this phenomena [16–18].

Our aim was to conduct a systematic review on values and preferences for LMWH therapy during pregnancy using a mixed-methods integrative design. We conducted this review as part of the DASH-TOP project [19] that aims to improve the quality of thromboprophylaxis decisions in this population.

## Methods

We registered the protocol in PROSPERO (CRD42020193925), and adhered to the PRISMA (Preferred Reporting Items for Systematic reviews and Meta-Analyses) 2020 statement [20].

### Design

**Fig. 1:**
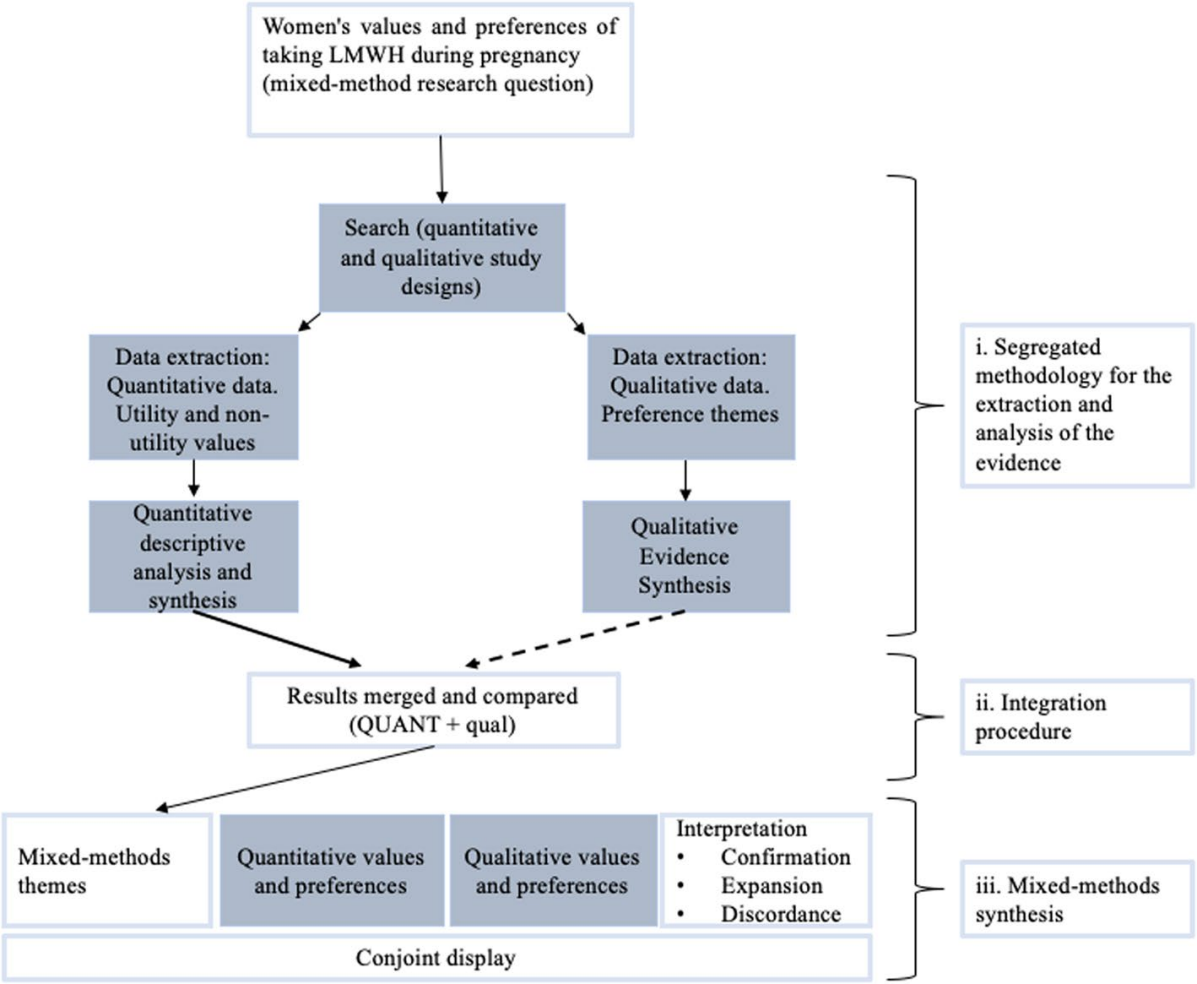
Mixed-method analysis and synthesis

We followed a mixed-methods approach [21–24] to synthesize and integrate different types of evidence, either quantitative and qualitative [25–27]. We followed a convergent integrated design that comprises three-steps (Fig. 1) i) Segregated data extraction and analyses of the evidence, maintaining a clear distinction between quantitative and qualitative datasets with individual synthesis prior to the mixed-methods synthesis [24, 28]; ii) Integration of data following a QUANT + qual integration procedure [25]; and, iii) Mixed-methods synthesis.

### Data sources and searches

We used a validated search strategy to identify studies on patients’ values and preferences [29]. The search strategy used terms related to LMWH and pregnancy. Searches were conducted from inception to September 2020 in the following databases: in MEDLINE (accessed via PubMed), PsycINFO (accessed via EBSCO host), CINAHL (accessed via EBSCO host), and The Cochrane Central Register of Controlled Trials (search strategies are available in the Additional file 1. and preserve search strings on searchRxiv https://searchrxiv.org/). We conducted literature surveillance via MEDLINE (accessed via PubMed alerts) until the review was submitted for publication (July 7^th^, 2022). We did not restrict our search by publication status, language, or date of publication. We also reviewed reference lists of the included articles, and relevant systematic reviews.

### Eligibility criteria

**Fig. 2:**
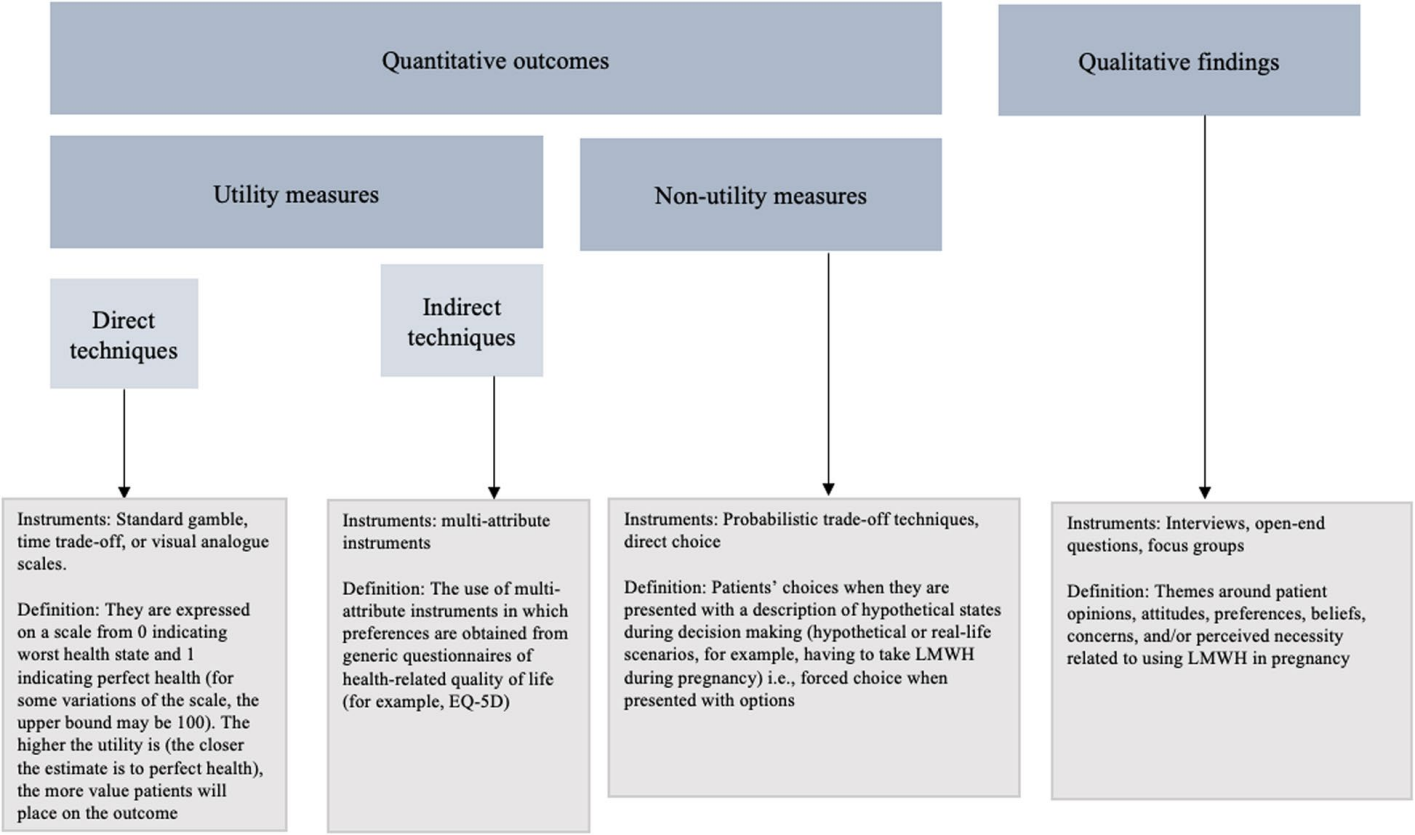
Preference-elicitation methods

We included studies that enrolled pregnant women, or women who were planning pregnancy, for whom anticoagulation with LMWH was considered and:

• Examined women’s values and preferences for LMWH vs. watchful waiting or alternative anticoagulant therapy.
• Examined choices patients make when presented with management options regarding antithrombotic therapy.
• Examined women’s experiences and beliefs of LMWH therapy in pregnancy.
- We considered studies to be eligible using preference-elicitation methods detailed in (Fig. 2) Selva.et.al. 2017 [13, 29].

We excluded studies that rated health states related to VTE in pregnancy, but did not involve the decision of whether to use LMWH. We also excluded studies addressing the use of other antithrombotic medicines such as aspirin. Finally, we excluded study protocols, conference abstracts, reviews, and non-peer reviewed publications such as letters or editorials.

### Selection of studies and data extraction

All steps were conducted independently by pairs of calibrated reviewers, using standardized and piloted forms. Disagreements were resolved with the help of a third reviewer.

We extracted information on—study design; objectives; population characteristics (mean age, level of education and income level); reasons for VTE risk in pregnancy (for example: history of VTE, thrombophilia, mechanical heart valves, antiphospholipid syndrome); if LMWH was used for prevention or treatment during antepartum, postpartum or both periods; and, a description of the methods used to obtain preferences (including instruments and techniques for preference elicitation). Outcomes included quantitative utility values and non-utility measures (collected as means with standard deviations, interquartile ranges or percentages, as available), and qualitative preferences (collected as themes and illustrative quotes).

### Data synthesis and analysis

We conducted a descriptive analysis for quantitative data [30]. For utility outcomes, we reported the mean value for the scores of the health state ‘Pregnancy with LMWH’ (participants rate how close the health state ‘pregnancy with LMWH’ is to good health on a 0–100 scale) [13, 31]. For non-utility outcomes, we reported overall means, frequencies or proportions.

For qualitative findings, we conducted open coding thematic analyses to collect and analyze the data [28, 32, 33]. We extracted interpretative findings reported by authors and supporting quotes. We categorized these findings in themes using an iterative process that involved a careful and repetitive reading of all pieces of extracted text.

For the mixed-method synthesis we integrated the data following a QUANT + qual design [25]: themes from the quantitative data were prioritized and supplemented with qualitative findings (the rationale for having the quantitative dataset leading the integration is that there is a larger body of evidence [29]). We presented this integration process using a conjoint display [22, 26, 27]; specifically, we used a side-by-side comparison [21, 22, 27] to assess whether datasets were in discordance, confirmation, or expansion [27]. Discordance was defined as quantitative and qualitative results that were inconsistent or contradictory. Confirmation occurred when findings from both types of data reinforced each other. When findings from one type of data expanded upon insights from the other type of data, this was classified as expansion. Findings were synthesized and reported narratively and tabulated.

### Appraisal of the evidence

For quantitative studies, we applied the GRADE approach to assess the risk of bias and certainty of evidence [34, 35]. For qualitative studies, we used the CASP Qualitative Checklist tool [36, 37] to appraise the methodological quality, and the CERQual (Confidence in the Evidence from Reviews of Qualitative Research) approach to assess the certainty of evidence [38].

To assess the certainty of the evidence of the mixed-methods findings, we selected the dataset with the highest certainty of evidence [39].

## Results

### Study and population characteristics

**Fig. 3:**
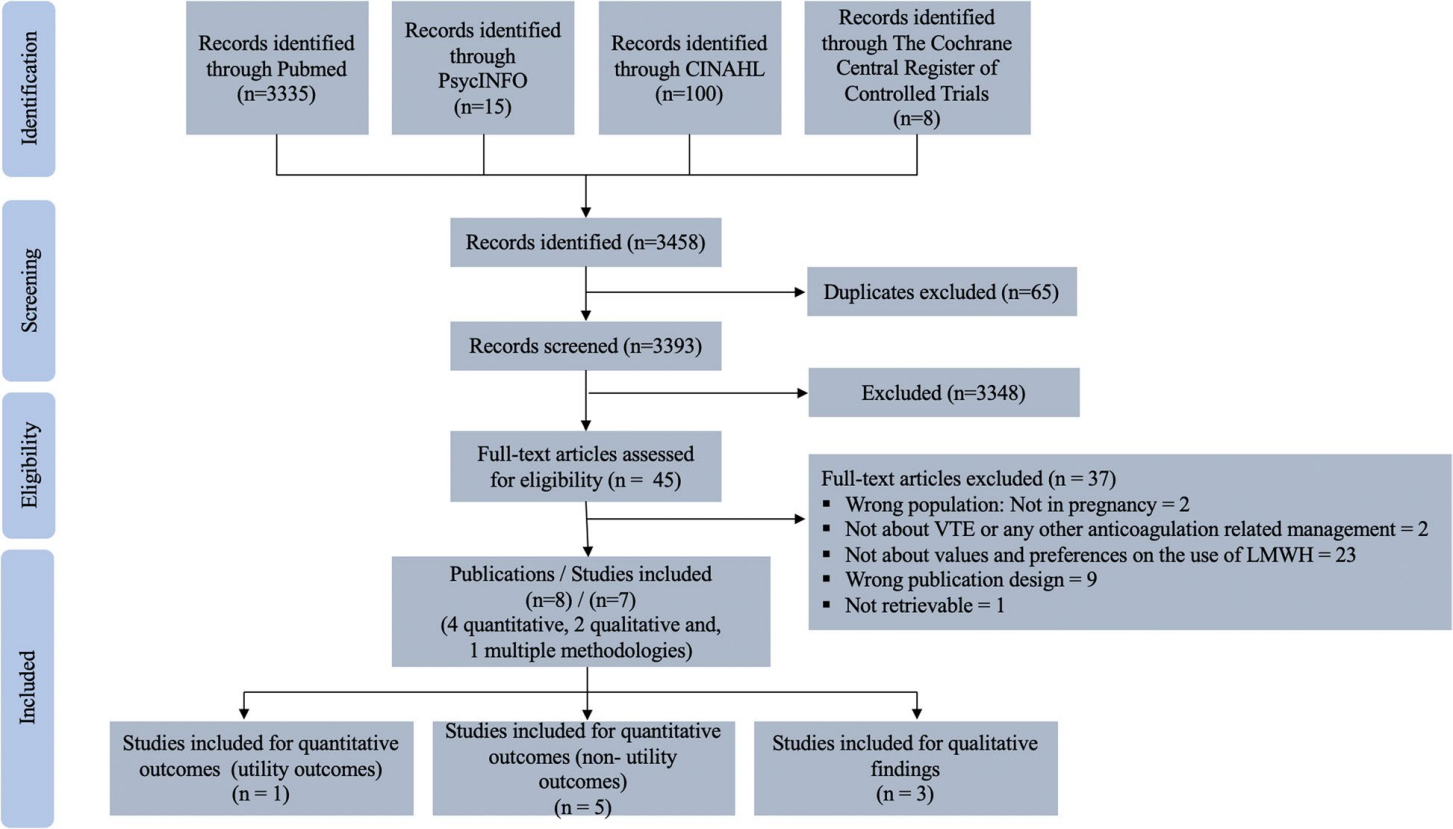
Preferred Reporting Items for Systematic Reviews and Meta-Analyses (PRISMA. Statement 2020) flow chart)

We identified 3,393 references, of which eight publications [40–47] reporting on seven studies met our eligibility criteria (Fig. 3).

Included studies are summarized in Table 1.

**Table 1:** Study and population characteristics

Author, Year, Country [reference]	Objective	Population						Methods		GRADE (Risk of Bias) /CASP (unmet methodological quality criteria)
		Participants, n	Mean age (years) (SD /range)	Education (%)	Race (%)	Pregnancy period, Reason for VTE risk in pregnancy	Indication for the use of LMWH	Study design [Data Collection Methods]	Outcome presentation: Measurement instrument	
Anderson, 1993 [43] Canada	To determine the following: (1) whether women requiring subcutaneous heparin therapy during pregnancy preferred administering the drug through an indwelling Teflon catheter as opposed to receiving twice daily subcutaneous injections and (2) the efficacy and feasibility of administering heparin through an indwelling Teflon catheter	12	28.6	NR	NR	Period: Ante-partum Reason: Previous VTE event	Prevention/ Treatment	Cross-sectional [Questionnaire]	Non-utility: Researchers self-developed questionnaire	Moderate risk of bias
Bates 2016 [41], Eckman 2015 [47], Multicenter: Canada, USA, Brazil, Finland, Norway and Spain	To compare women's choices regarding thromboprophylaxis during pregnancy	123	33.94 (6.2)	(1) Not complete high school: 13.8% (2) Completed high school: 18.7% (3) Some post-secondary or higher: 67.5%	NR	Period: Ante-partum Reason: Previous VTE event	Prevention	Cross-sectional [Value elicitation exercises and Direct choice exercise]	Utility: Direct techniques: VAS Non-utility: Direct choice exercise	Moderate risk of bias
Guimicheva 2019 [40] UK	To explore women's views, experiences and adherence to postnatal thromboprophylaxis	67	36.2 (4.4)	NR	Caucasian (73.1) African-Caribbean (23.9) Other (3.0)	Period: Post-partum Reason: Previous obstetric complications	Prevention	Cross-sectional [Questionnaire]	Non-utility: BMQ- questionnaire	Serious risk of bias
Hordern, 2015 [42] UK	To assess reported patient compliance with a standard course of postnatal thromboprophylaxis in the form of low-molecular-weight heparin (LMWH)	111	NR	NR	NR	Period: Post-partum Reason: Multiple risk factors for VTE: Previous VTE event, unprecedented thrombophilia thrombosis, and previous obstetric complications	Prevention	Cross-sectional [Structured interview]	Non-utility: Researchers self-developed questionnaire	Serious risk of bias
Martens, 2007 [45] Canada	To explore the unique experiences, challenges, and coping strategies of pregnant women diagnosed with thrombophilia and who are on daily heparin injections	9	30 to 36	NR	NR	Period: Ante-partum and post-partum Reason: Unprecedented thrombophilia thrombosis	Prevention	Qualitative [Semi-structured interview]	Qualitative: Analytical themes	4 and 6^a^
Patel 2012 [44] UK	To monitor women's adherence to enoxaparin when prescribed during pregnancy and the puerperium and to explore women's views and beliefs about the enoxaparin prescribed	95 (quantitative) 30 (qualitative)	33.03 years (range 18–46)	NR	Caucasian (56.8) African-Caribbean (31.6) Asian (4.2) Other (7.4)	Period: Ante-partum and post-partum Reason: Multiple risk factors for VTE: Previous VTE event, unprecedented thrombophilia thrombosis, and previous obstetric complications	Prevention/ Treatment	Multiple methodology [questionnaire with open-ended questions]	Non-utility: BMQ- questionnaire Qualitative: Open-ended questions	Moderate risk of bias
Skeith 2021 [46] Canada	To understand how patients and physicians navigate the decision-making process for use of LMWH and/or ASA in pregnancy	10	NR	NR	NR	Period: Ante-partum and post-partum Reason: Unprecedented thrombophilia thrombosis and previous obstetric complications: at least 1 prior late pregnancy loss (> 10 weeks gestation) or 2 early pregnancy losses (< 10 weeks gestation)	Prevention	Qualitative [Semi-structured interview]	Qualitative: Analytical themes	4 and 6^a^

^a^ Criteria 4 and 6 correspond to CASP instrument criteria: selection bias and lack of consideration of the relationship between researchers and participants, respectively. NR: Not reported

From the studies, we included information on 427 women, with a mean age of 33.8 years (SD = 5.53). Two studies were focused on the antepartum period [41, 43, 47] in women where the risk of VTE was exclusively due to history of VTE: one compared their choices regarding thromboprophylaxis [41, 47]; the other [43] studied the use of unfractionated heparin (UFH) for both prevention and treatment purposes; although UFH was not part of our inclusion criteria we included this study as the authors reported on the preference regarding injectable administration of heparin, and in this regard we found it relevant to inform the context of this decision-making process. Two studies [40, 42] focused on the postpartum period for women with multiple risk factors for VTE. In both cases, authors explored women's views, experiences and adherence to LMWH for VTE prevention alone. The other three studies [44–46] assessed preferences, both during the antepartum and postpartum periods: two [45, 46] included women with antiphospholipid syndrome, which is associated with recurrent pregnancy loss. The other study [44], included women with multiple risk factors such as history of VTE, antiphospholipid syndrome, stillbirth in previous pregnancy and placental complications. They collected views and beliefs about enoxaparin prescribed for both prevention and treatment.

### Women’s values and preferences

One of the included studies reported utility measures [41, 47], five reported non-utility measures [40–44, 47], and three studies informed qualitative findings [44–46].

Women’s preferences reported from quantitative studies are outlined in Table S1, and from qualitative studies in Table S2 (available in Additional file 2).

#### Quantitative outcomes

##### Utility measures:

**Pregnancy with LMWH prophylaxis**

In women with a previous VTE event, the utility value for the health state ‘pregnancy with LMWH prophylaxis’ was measured using a feeling thermometer (a visual analogue scale (VAS)) [41, 47]. The overall mean and standard deviation (SD) was 81 [15], meaning that women placed receiving LMWH injections during pregnancy at around 80 on a scale of 0 to 100, hence, taking LMWH during pregnancy as something relatively close to good health.

##### Non-Utility measures:

**Willingness to take LMWH**

One study [41, 47] assessed the willingness for thromboprophylaxis with LMWH in the antepartum population (in women with a previous VTE event) using two different instruments:

i) A direct choice exercise in which authors presented women with a decision board that described different risks of developing VTE during pregnancy based on characteristics of their prior VTE. The results showed that the majority of women were willing to take LMWH, regardless of their VTE risk, and that the higher their risk, the greater was their willingness to take it.
ii) A probability trade-off exercise in which authors used this instrument to determine women’s thresholds for accepting LMWH prophylaxis [41]. The median threshold reduction in VTE risk at which women were willing to accept use of LMWH was 3%. Furthermore, women with less previous (under 2 weeks) experience with LMWH during pregnancy compared to those with more experience, required a greater VTE risk reduction; it also showed that there were no significant differences between pregnant women and women planning pregnancy.

Another study [42] assessed compliance with thromboprophylaxis in women with thrombophilia, and reported that if thromboprophylaxis was indicated in a future pregnancy, most women (94.5%) would accept it.

**Beliefs towards the harms, overuse, necessity, and concerns of taking LMWH**

Two studies [40, 44] used the Beliefs about Medicines Questionnaire (BMQ) [48] to assess the perception of using LMWH in women in the postpartum period who have presented a previous obstetric complication [40] or had multiple risk factors for VTE [44]. The BMQ questionnaire includes 18-items and four subscales assessing beliefs associated with the medication (in this case use of enoxaparin). Subscales for general harm and overuse (based on four items each) could have a minimum score of four and a maximum score of 20. Subscales for specific necessity and concerns (based on five items each) have a minimum score of five and a maximum score of 25.

The Necessity-Concerns Differential (NCD) [48], is used to report on the balance between necessity and concerns of medications. The Necessity-Concerns Differential (NCD) was found to be 1.18 [40] and 2.20 [44], both with a positive differential, suggesting that women felt that the necessity of the enoxaparin was greater than any concerns they may have held regarding its use.

**Reason for not being adherent when using LMWH**

One study [42] conducted in women who had multiple risk factors for VTE in postpartum, reported reasons for not completing treatment that included bruising or wound complications; forgetting; fear or dislike of needles, and emotional reasons for stopping.

**Preference for route of administration**

One study [43] assessed the preferred mode of administration for UFH in women with a previous VTE event. The majority preferred injecting heparin through a Teflon catheter over standard subcutaneous injections. This was due mainly because it caused less pain and less bruising than injections.

**Preferred amount of information regarding LMWH**

One study [42] assessed preferences regarding the amount of the information given about the decision to use LMWH during postpartum in women who had multiple risk factors for VTE in postpartum: the majority of women reported that they had received enough information about treatment, however 16.6% reported that they would have liked more information or training on injections before leaving the hospital.

#### Qualitative findings

Qualitative findings included results mainly from women with unprecedented thrombophilia, but one of the studies also included other risk factors for VTE [44]. We synthesized qualitative findings using five main themes.

**Attitude towards LMWH during pregnancy**

The majority of women were willing to receive LMWH injections [44–46]. They felt that responding to their situation by taking action with daily LMWH injections comforted them and decreased anxiety. Women understood that the desired outcome of a successful pregnancy could not be predicted and they accepted uncertainty and maintained the perspective that a positive outcome far outweighed any temporary discomfort. Miscarriage was considered a very traumatic situation and was perceived as leading to a high risk of complications during pregnancy, thus they felt motivated to use LMWH; in the study [44] that included women at low risk for VTE, participants still reported anxiety relief by using LMWH.

**Experience of using LMWH during pregnancy**

Three studies [44–46] reported challenges with injections, such as bruising, pain, and bleeding; one study [44] stated that easier routes of administration would be desirable. However, complications of the medication did not influence the decision to take LMWH, and it became part of their pregnancy experience.

**Concerns about medication**

The main concern surrounding LMWH was safety [44–46]. The majority of women placed a higher priority on their baby’s safety. However, in one study first-time mothers placed a higher priority on the baby’s safety, while mothers with other children prioritized their own health. Other concerns included fear of forgetting a dose; fear of needles; fear of bleeding during labor; and needing a scheduled labor (off LMWH) to be able to get an epidural.

**Information needs to inform the decision**

Two studies [44, 45] found that the majority of women felt they had not received enough information to address their concerns. Most frequently, women felt uninformed about how LMWH worked, injection technique and side effects. The information was not always prompted by the physician and, in many cases, there were limited available resources for women. Information was gathered from different sources including the internet, books on pregnancy, health professionals, or word of mouth. Sharing stories with other women proved to have a compelling influence on decision making, and was also reassuring and encouraging.

**Patient involvement in the decision-making**

Seeking information proved a powerful means by which women were able to take control and actively address their needs. In addition, patients described how physicians influenced their decision-making process, highlighting the importance of the physician–patient relationship [44–46]. Patients felt empowered by their healthcare professionals and did not feel pressured to take LMWH. Several women [45] expressed anxiety regarding the weight of responsibility involved in making medical decisions that could affect the pregnancy outcome.

### Mixed-methods results

Three quantitative outcomes (Beliefs about harms, overuse, necessity, and other concerns about taking LMWH; reasons for reason for not being adherent when using LMWH; preference for route of administration) were merged into a single outcome to inform the corresponding mixed-methods theme (“Beliefs towards medication”). Four mixed-methods themes were identified. Results are detailed in Table 2.

**Table 2:** Conjoint display

**Mixed-methods themes [ref]**	**Quantitative outcomes**		**Qualitative findings**		**Mixed-methods findings** *(Confirmation, Expansion, Discordance)*	Certainty of the evidence
Pregnancy with LMWH prophylaxis [Bates 2016 [41], Eckman2015 [47]; Skeith 2021 [46]; Martens 2007 [45]; Patel 2012 [44]]	Utility Value: Mean VAS scale (0–100) [ref]	Certainty of evidence	Theme(s) [ref], *representative quote*	Certainty of evidence		
	[Bates 2016 [41], Eckman 2015 [47]]	Very Low	Attitude towards the decision -making of using LMWH [Skeith 2021 [46]; Martens 2007 [45]; Patel 2012 [44]] *LMWH injections with “minimal side effects, and that, compared to the emotional pain with loss, a little bit of physical pain from a needle is small potatoes*]	Moderate	***Confirmation*** Both datasets confirm the relief of having LMWH as an option far outweighed any temporary discomfort caused by the injections, and reduced anxiety towards the disease	Moderate
			Experience of using LMWH during pregnancy [Skeith 2021 [46]; Martens 2007 [45]; Patel 2012 [44]] *Although it wasn’t fun injecting myself it was part of the ritual. …It felt like I was doing something instead of just waiting there to see if I would miscarry. …It felt like I at least had a very, very, very small hand in helping]*	Moderate		
Willingness to take heparin [Bates 2016 [41], Eckman 2015 [47]; Hordern 2015 [42]; Skeith 2021 [46]; Martens 2007 [45]; Patel 2012 [44]]	Non-utility: Proportion (%) of women willing to take heparin (instrument [ref])	Certainty of evidence	Theme(s) [ref], *representative quote*	CERQUAL assessment		
	-78.86 (direct choice) [Bates 2016 [41], Eckman2015 [47]] -94.5 (RSQ^b^) [Hordern 2015 [42]]	Very Low Very Low	Attitude towards the decision -making of using LMWH [Skeith 2021 [46]; Martens 2007 [45]; Patel 2012 [44]] *When you want to have a baby … nothing will stop you* *Definitely gives me piece of mind during pregnancy; without it I would feel very nervous about developing another DVT*	Moderate	***Expansion*** Women accepted uncertainty regarding the pregnancy outcome and were willing to take heparin as it was felt they were taking- action on the management of their condition. Most of the women from the qualitative data were using LMWH to prevent pregnancy loss, hence higher risk perception than when used to prevent DVT (as shown by the non-utility values; women at lower risk were less willing to take the medication). Risk perception is correlated with willing to take LMWH	Moderate
	Non-utility: Median (%) of risk reduction of LMWH to be willing to take (instrument [ref])					
	3 (probability trade-off) [Bates 2016 [41], Eckman 2015 [47]]	Very Low				
Beliefs towards medication [Guimicheva 2019 [40]; Patel 2012 [44]; Hordern 2015 [42]; Anderson 1993 [43]; Skeith 2021 [46]; Martens 2007 [45]; Patel 2012 [44]]	Non-utility: Mean (± ^a^) Necessity- concerns differential (instrument [ref])	Certainty of the evidence	Theme(s) [ref], *representative quote*	CERQUAL assessment		Moderate
	+ (BMQ- Scale) [Guimicheva 2019 [40]; Patel 2012 [44]]	Very Low	Concerns about medication [Skeith 2021 [46]; Martens 2007 [45]; Patel 2012 [44]] *I wanted a baby so bad, I was like I don’t care, I’ll do it… the chance of it harming me…*	Moderate	***Confirmation*** Patients focused on safety issues. Women place a higher priority on the impact LMWH has on the unborn baby compared with any impact the medicine may have on them	
	Non-utility Values: Reasons for not being adherent when using LMWH (% of women) (instrument [ref])	Certainty of the evidence	Theme(s) [ref], *representative quote*	CERQUAL assessment		
	-Bruising (25 (RSQ^b^) [Hordern 2015 [42]] -Forgetting (16.6(RSQ^b^ [Hordern 2015 [42]] -Fear or dislike of needles (16.6(RSQ^b^) [Hordern 2015 [42]]	Very Low	Concerns about medication [Skeith 2021 [46]; Martens 2007 [45]; Patel 2012 [44]] *So, then you’ re worried because labor it isn’ t always planned, right, and what if … I mean what if I went into labor at 33 [weeks] and I took [LMWH] yesterday or I took it today and I would worry that I would go into labor; what would ultimately happen, being on the medication and going into labor*	Moderate	***Expansion*** Other concerns provided by the qualitative data were regarding withholding injections before delivery and a scheduled labor	Moderate
	Non-utility Values: Preference for route of administration (Instrument, % of women) ([ref])	Certainty of the evidence	Theme(s) [ref], *representative quote*	CERQUAL assessment		
	Teflon catheter (RSQ^b^) = 83.3 [Anderson 1993] [43]	Very Low	Experience of using LMWH during pregnancy [Skeith 2021 [46]; Martens 2007 [45]; Patel 2012 [44]] *If there was a way to get injections for enoxaparin *via* an epipen type device it would be much more tolerable*	Moderate	***Confirmation*** Women from both datasets agreed that they preferred devices that would facilitate administration of injections	Moderate
Preferred amount of information regarding LMWH [Hordern 2015 [42]; Skeith 2021 [46]; Martens 2007 [45]; Patel 2012 [44]]	Non-utility Values: Adequate information regarding LMWH (Instrument, % of women) ([ref])	Certainty of the evidence	Theme(s) [ref], *representative quote*	CERQUAL assessment		
	Whether woman had received enough information regarding LMWH (RSQ^b^) = 83.8 [Hordern 2015 [42]]	Very Low	Information needs to inform the decision [Martens 2007 [45]; Patel 2012 [44]] *I have no issues injecting if it is safeguarding mine and the baby’s health but I lack some faith in the safety/side-effects/ general effects of the medicine. Published information on Clexane seems to be contradictory*	Low	***Discordance*** While quantitative data showed that patients felt well-informed about their decision; in qualitative data the majority of women felt they had not received enough information to address their concerns. Women expressed feeling confused about how VTE could affect their baby, how it could compromise their own health and why it was particularly relevant during pregnancy	Low
			Patient involvement in the decision-making [Skeith 2021 [46]; Martens 2007 [45]; Patel 2012 [44] ] *I do not have a problem doing injections and was aware of the possibility of the injections before becoming pregnant. However, I think other women might benefit from more time and support around the use of Clexane in their pregnancy*			
			Patient involvement in the decision-making [Skeith 2021 [46]; Martens 2007 [45]; Patel 2012 [44]] *I felt pretty involved. I didn’t feel like pressured into taking [LMWH] if I did get pregnant. It was really up to me to say I want to take the injections or not… I felt involved in the decision*	Moderate	***Expansion*** Qualitative data informed that women felt involved in the decision-making; healthcare professionals are an important role in support the decision-making process	Moderate
	Would have liked more information or training before leaving hospital (RSQ^b^) = 16.6 [Hordern 2015 [42]]	Very Low	Information needs to inform the decision [Martens 2007 [45]; Patel 2012 [44]] *No, I don’t remember being given any information and you are insecure about the whole subject, so you are not prepared to ask questions because you don’t know what to ask*	Low	***Discordance*** Datasets are in discordance in whether it was an informed decision-making process. Injection administration technique was an important need of information	Low

^a^ ( ±): Positive values ratio means that women place higher value to the necessity of medication than concern; for negative values it would be the opposite (BMQ scale)

^b^*RSQ* Researchers self-developed questionnaire

#### Pregnancy with LMWH prophylaxis

This theme was informed by four studies [41, 44–47]. Both datasets confirm findings on considering this health state as close to ‘perfect health’ and having LMWH as an option far outweighed any temporary discomfort caused by the injections. Prior experience using LMWH was important and very informative on how women considered daily injections.

#### Willingness to take heparin

Five studies informed this theme [41, 42, 44–47]. Both data sets confirmed that the majority of women would be willing to take LMWH; that they understood that the desired outcome of a successful pregnancy could not be predicted, and uncertainty was well tolerated. The quantitative data expands on this by showing a direct relationship between high perceived risk and increased willingness to take the medication.

#### Beliefs towards medication

This mixed-method theme was reported by six studies [40, 42–46]. There was confirmation that women viewed LMWH more as a necessity than a concern; the main consideration being safety, especially for their unborn baby. Women preferred to use devices that facilitated the administration of the injections. Qualitative data expanded upon quantitative data, by reporting other concerns associated with antepartum use of LMWH (for example, withholding injections before a scheduled labor and delivery).

#### Preferred amount of information regarding LMWH

This mixed method theme was the least informed. Three studies reported discordances [42, 44, 45] and four included expansion [42, 44–46]. Discrepancies occurred when women were asked about their preferences for the amount of information they received. Quantitative results reported that women felt well-informed, while the opposite was the case in qualitative findings.

In qualitative reports women understood that benefits outweighed risks, but they didn’t feel they had sufficient information, especially about the effect of LMWH on their condition, injection techniques, side effects, or what to do if a difficult situation arose. Qualitative data also expanded findings, showing that this decision needs adequate support from the healthcare professional.

### Quality appraisal

Assessments of the quality of the evidence are available Additional file 3. The certainty of the evidence for all the quantitative outcomes was rated as very low, mainly due to risk of bias (unclear sampling strategies [41, 43, 44, 47] and high attrition rates [40, 42]); indirectness (due to methodological elements [40–44, 47]); and imprecision (small sample sizes [40–44, 47]). Regarding qualitative research, both studies [45, 46] presented methodological concerns regarding selection bias, and lack of consideration of the relationship between researchers and women. The confidence for all qualitative findings was moderate (due to concerns about rigor (unclear recruitment and sampling strategy)) except for the finding Information needs to inform the decision [44, 45], which was rated as low, due to concerns regarding adequacy and relevance of the data. The certainty of the evidence for all the mixed-methods themes was rated as moderate, except for ‘Adequacy of the information regarding LMWH’ which was low.

## Discussion

### Main findings

Our mixed methods systematic review is the first to assess preferences of women towards using LMWH during pregnancy. We included seven studies: five studies [40–44, 47] were conducted among women who had a history of VTE, in which heparin was used for VTE prevention and/or treatment in the ante- and/or post-partum period. The other two qualitative studies [45, 46] were conducted in women with thrombophilia, where LMWH was used to reduce risk of miscarriage.

After quantitative and qualitative datasets were merged, four mixed- methods findings were identified reporting on women’s preferences towards using LMWH during pregnancy.

For all included women (women with a previous VTE event, unprecedented thrombophilia thrombosis, and previous obstetric complications), datasets confirmed each other in that: 1) the majority of women considered that benefits of treatment outweighed the inconvenience of daily injections; and 2) main concerns were about medication safety and the need to give injections. Quantitative outcomes expanded on the qualitative findings in that: 3) women who perceived a higher risk of VTE (those who had an unprovoked previous VTE and those with thrombophilia) were more willing to take LMWH. Finally, we found a discrepancy between the datasets regarding: 4) the amount of information preferred to make the decision; however, qualitative data expanded to clarify this discrepancy. Women expressed feeling confused about how VTE could affect their baby, how it could compromise their own health, and why it was particularly relevant during pregnancy. Therefore, informed decisions are preferred and the role of clinicians to support their decision-making process was highlighted for women with multiple risk factors for VTE: previous VTE event, unprecedented thrombophilia thrombosis, and previous obstetric complications.

The overall quality of the evidence was moderate.

### Limitations, strengths and previous research

Our review has several limitations. There are still very few studies, including very few women, in this field. As a result of this limitation in the body of evidence, we only captured preferences for women at risk for VTE or pregnancy loss during pregnancy. We were not able to identify preferences in other conditions such as women with heart valve prostheses [8, 9, 49, 50]. In addition to the limited number of published studies, another reason to downgrade the quality of the evidence in our review is inconsistency across studies. For example, for outcomes such as “willingness to take LMWH” the patient populations included women using LMWH to prevent miscarriage (women with thrombophilia) and women using it as thromboprophylaxis to prevent recurrent VTE. Risk perceptions differ according to the condition for which LMWH was going to be used for. This was evident in one of the studies that included both populations; those with thrombophilia had higher risk perception vs those with a prior VTE [44]. Thus, we were unable to meta-analyze preferences for each population group: previous VTE event, unprecedented thrombophilia thrombosis, and previous obstetric complications. Despite these limitations, by leveraging both qualitative and quantitative data, our review was able to demonstrate a relationship between level of VTE risk and willingness to take LMWH.

Another limitation of our review was the lack of consideration for other VTE treatment options like aspirin (ASA), which is recommended by various obstetrician societies [8, 51–53] to reduce obstetric complications such as preeclampsia. Despite that, we included only LMWH because it is the unique anticoagulant showing safety for the baby as it does not cross the placenta. The encouraged use of the combination LMWH with ASA [51, 53] by obstetricians should be noted and future research should address women’s preferences for this combination, especially in women at high risk [9, 52].

As noted in other reviews of values and preferences [13, 54], we were able to collect and analyze quantitative data more easily than qualitative data. As measures are inconsistent and we lack a specific framework to guide the analysis of qualitative data, we opted to use an open coding approach for extracted data, which is the gold standard methodology to study phenomena in qualitative methods [28, 33]. In addition, we used a validated search strategy that was designed to include qualitative studies containing preferences [29] to ensure the identification of the full body of qualitative evidence. Therefore, a strength of this systematic review was our demonstration that qualitative methods are useful to inform on the context, the grounds by which the decision is made; and address issues like level of information is needed to inform the decision, what is the preferred level of patient involvement, and the role of the healthcare professional in supporting the decision [46].

Assessing the certainty of the evidence is critical in understanding how our findings support suggestions to use shared decision-making in clinical practice and guideline development. Although, there are specific quality of evidence appraisal tools for mixed- methods systematic reviews, these methods still face the challenge of assessing integrated findings [55]; hence, we assessed independently the certainty of the evidence for both datasets using the GRADE approach, which has specific guidance for the topic. We used specific guidelines to assess the evidence about values and preferences described in quantitative findings [34, 35], and used GRADE-CERqual to judge qualitative evidence. [34, 35, 56]. Finally, we selected findings with the highest certainty to inform the quality of our mixed- methods findings [39].

An important strength of this review is the mixed-methods integrative approach, which expanded our findings, by increasing the ability of data analyzed and subsequently used to inform policy and practice [25]. The design of this review leverages qualitative and quantitative data to help us confirm and expand on findings, as well identify discordances between the types of data. Other mixed-methods systematic reviews [13, 54, 57] assessing preferences using different methods to analyze outcomes, rarely integrated data from quantitative and qualitative datasets to improve understanding of the phenomena [25]. The conduction of an integrative analysis contributes to the field of mixed methods research [15] and specifically in the field of systematic reviews [28, 58]. Also, attempting to integrate data and findings from the individual components is considered adequate criteria in Good Reporting of A Mixed Methods Study (GRAMMS) [14].

### Implications for practice and research

Venous thromboembolism is recognized as a leading cause of maternal death in high income countries and the use of LWHM is the gold standard preventive and treatment strategy [10, 59]. However, the efficacy of LMWH in pregnancy continues to be uncertain, mainly due to the high rate of refusals in RCT [60–62] over 20% of the included participants. Clinicians included in these trials showed to be dedicated, well-informed, and experienced counselors. However more research is needed on women’s preferences for VTE prophylaxis during pregnancy (specially in high risk and for preventive purposes) to support this clinical decision-making. Our findings can support recommendations [12, 52] regarding the types of information pregnant women at risk of VTE need to participate in a SDM process and raise awareness among obstetricians and anesthesiologists that potential risks are high and prepare them for timely adaptation of medication when necessary [10]. These factors have been shown to influence the decision-making process [46], and their clarification is especially important in low risk settings (i.e., women with a prior history of VTE associated with a non-hormonal temporary provoking risk factor), in which we showed lower levels of willingness to take antithrombotic treatment [41, 47]. This is particularly relevant when using systematic reviews to inform the development of tools used to support SDM [19].

More research is needed on specific qualitative frameworks to assess preferences delivered through qualitative instruments, such as interviews or clinical observations [63]. For example, one study [46] included in this review reported that the husband of a pregnant women was very concerned about the safety of LMWH and its effect on his wife’s health. ‘What other people think I should do’ is an aspect that can affect the decision. Burke’s motives pentad framework was used to deductively categorize patient reflections by their reasons, as to why their care plans made sense in the context of thromboprophylaxis in atrial fibrillation decision-making [64].

In addition, further guidance from the GRADE-CERQUAL group is also needed to assess certainty of the evidence in mixed methods reviews that integrates findings coming from different methodologies. The CERQUAL methodology to assess the relevance and adequacy domains [65, 66] may help clarify when findings coming from different study designs are complementary or discordant among studies [38].

## Conclusion

This mixed-method systematic review showed among women at risk for recurrent VTE during pregnancy and pregnancy loss, LMWH prophylaxis was preferred to watchful waiting due to its perceived net clinical benefit. However, more evidence is needed in women at lower risk of VTE in pregnancy as the certainty of this evidence was only moderate. Integrating data from different sources of evidence, and representing them in a joint manner helps us better understand women’s preferences and contributes to the field of mixed-methods research. Our results may inform clinical practice guidelines and support a shared decision-making process in the clinical encounter for the management of VTE in the context of pregnancy.

## Supplementary Information

**Additional file 1.**

**Additional file 2.**

**Additional file 3.**

## Data Availability

All data generated or analysed during this study are included in this published article [and its supplementary information files]. Preserve search strings are available in searchRxiv https://searchrxiv.org/).
