# Supplementary material for: Women’s values and preferences on low-molecular-weight heparin and pregnancy: a mixed-methods systematic review
Source: BMC Pregnancy Childbirth. 2022 Oct 5;22:747. doi: 10.1186/s12884-022-05042-x (PMC9533610; doi:10.1186/s12884-022-05042-x)
Supplement: Supplementary file 1 — Additional file 1. [file 12884_2022_5042_MOESM1_ESM.docx]

1. Search Strategy

1.1 MEDLINE (accessed via Pubmed)

(((Pregnan*[tiab] OR gesta*[tiab] OR placenta*[tiab] OR "pregnancy" [MeSH] OR "Pregnant Women" [MeSH]) AND (LMWH [tiab] OR low-molecular-weight-heparin[tiab] OR heparin*[tiab] OR prophylaxis [tiab] OR "Heparin, Low-Molecular-Weight" [MeSH])) AND (("Attitude to Health"[MeSH] OR "Patient Participation"[MeSH] OR "Patient Preference"[MeSH] OR preference*[tiab] OR choice*[ti] OR value*[ti] OR health state value*[tiab] OR valuation*[ti] OR expectation*[tiab] OR attitude*[tiab] OR acceptab*[tiab] OR knowledge[tiab] OR point of view*[tiab] OR user* participation[tiab] OR patient* participation[tiab] OR patient* perspective*[tiab] OR user* perspective*[tiab] OR patient perce*[tiab] OR patient* perce*[tiab] OR health perception*[tiab] OR user* perce*[tiab] OR user* view*[tiab] OR patient* view*[tiab] OR knowledge[tiab]) OR ((health[ti] AND utilit*[ti]) OR gamble*[tiab] OR prospect theory[tiab] OR preference score[tiab] OR preference elicitation[tiab] OR health utilit*[tiab] OR utility value*[tiab] OR utility score*[tiab] OR utility estimate*[tiab] OR health state[tiab] OR feeling thermometer*[tiab] OR best-worst scaling[tiab] OR VAS OR visual analogue scales OR standard gamble[tiab] OR time trade-off[tiab] OR TTO[tiab] OR probability trade-off[tiab] OR utility score[tiab] OR preference based[tiab] OR preference score*[tiab] OR "Decision Support Techniques"[MeSH]) OR (EQ 5D[tiab] OR EQ5D[tiab] OR EuroQol 5D[tiab] OR EuroQol5D[tiab] OR euroQoL[tiab] OR SF6D[tiab] OR SF 6D[tiab] OR Quality of Life[tiab] OR health utility index[tiab] OR HUI[tiab] OR 15D[tiab] OR patient-reported outcomes measurement information system[tiab] OR PROMIS-29[tiab] OR multiattribute[tiab] OR multi attribute[tiab] OR "Health Status"[MeSH]) OR ((((decision*[ti] AND maki*[ti]) OR decision mak*[tiab] OR decisions mak*[tiab]) AND (patient*[tiab] OR user*[tiab])) OR decision tool*[tiab] OR decision board*[tiab] OR decision analy*[tiab] OR decision tree[tiab] OR decision-support[tiab] OR decision aid*[tiab] OR direct choice*[tiab] OR discrete choice*[tiab] OR choice*[tiab] OR (survey*[tiab] OR questionnaire*[tiab]) OR ("Decision Making"[MeSH] AND (patient*[ti] OR user*[ti])) OR "decision support techniques"[MeSH] OR "Surveys and Questionnaires"[MeSH]) OR (interview*[tiab] OR focus group*[tiab] OR "Data collection" [MeSH])))

- 1. PsycINFO and CINAHL (accessed via EBSCO host)

| S1 | AB (pregnancy or pregnant or prenatal or antenatal or perinatal or maternal ) OR AB ( pregnant women or pregnant woman or pregnancy or pregnant or expecting mother ) OR AB ( pregnancy outcomes or pregnancy complications ) OR AB placenta |
| --- | --- |
| S2 | MH pregnancy OR MH pregnancy complications |
| S3 | S1 OR S2 |
| S4 | AB ( attitudes or perceptions or opinions or thoughts or feelings or beliefs or views or experience ) OR AB preference assessment OR AB ( preferences or attitudes ) OR AB preferential treatment OR AB ( choices or decision making ) OR AB choice boards OR AB ( perspectives or views or perceptions or attitudes or opinion ) OR AB ( perspective or perception or opinion or experience or attitude or views or thoughts or feeling ) OR AB ( participation or engagement or involvement ) OR AB health state OR AB ( knowledge or education or understanding or awareness ) |
| S5 | MH perception OR MH preferential treatment OR MH (views or opinions or perceptions or beliefs or attitudes or experience) OR MH (perspective or perception or opinion or experience or attitude) OR MH expectations OR MH (knowledge or education or understanding or awareness) OR MH value based healthcare OR MH (values and beliefs ) OR MH value based care OR MH value capture OR MH ( participation or engagement or involvement ) OR MH preference assessment |
| S6 | S4 OR S5 |
| S7 | AB health utility index OR AB preference-based measure of hrqol OR AB utility OR AB visual analogue scale OR AB standard gamble OR AB time trade-off OR AB trade off OR AB best-worst scaling OR AB decision support system OR AB decision support OR AB decision analysis OR AB decision analysis for management judgment |
| S8 | MH health utility index OR MH preference-based measure of hrqol OR MH utility OR MH visual analogue scale OR MH standard gamble OR MH time trade-off OR MH trade off OR MH best-worst scaling OR MH decision support system OR MH decision support OR MH decision analysis OR MH decision analysis for management judgment |
| S9 | S7 OR S8 |
| S10 | AB (decision making or decision-making or decision making process or decision-making process) OR AB decision making process OR AB decision tree OR AB (decision aid or decision tool ) OR AB decision support techniques OR AB decision support tool OR AB informed decision making OR AB informed choice OR AB decision making capacity |
| S11 | MH (decision making or decision-making or decision making process or decision-making process) OR MH decision making process OR MH decision tree OR MH ( decision aid or decision tool ) OR MH decision support techniques OR MH decision support tool OR MH informed decision making OR MH informed choice OR MH decision making capacity |
| S12 | S10 OR S11 |
| S13 | AB interview OR AB (survey or questionnaire) OR AB ( survey or questionnaire or instrument or measure or assessment or scale ) OR AB surveys for data collection OR AB data collection methods in qualitative research OR AB focus group OR AB focus group interview OR AB focus groups research method |
| S14 | MH interview OR MH (survey or questionnaire) OR MH (survey or questionnaire or instrument or measure or assessment or scale ) OR MH surveys for data collection OR MH data collection methods in qualitative research OR MH focus group OR MH focus group interview OR MH focus groups research method |
| S15 | S13 OR S14 |
| S16 | AB low molecular weight heparin OR AB (heparin or anticoagulants or heparin dose) OR AB heparin therapy OR AB heparin injection |
| S17 | MH low molecular weight heparin OR MH (heparin or anticoagulants or heparin dose) OR MH heparin therapy OR MH heparin injection |
| S18 | S16 OR S17 |
| S19 | S6 OR S9 OR S12 OR S15 |
| S20 | S3 AND S18 AND S19 |

- 1. The Cochrane Central Register of Controlled trials

| #1 | (pregnan*):ti,ab,kw |
| --- | --- |
| #2 | MeSH descriptor: [Pregnancy] explode all trees |
| #3 | #1 OR #2 |
| #4 | MeSH descriptor: [Heparin, Low-Molecular-Weight] explode all trees |
| #5 | ("LMWH"):ti,ab,kw OR ("low molecular weight heparin"):ti,ab,kw OR ("heparin"):ti,ab,kw |
| #6 | #4 OR #5 |
| #7 | #3 AND #6 |
| #8 | (attitude):ti,ab,kw OR ("participation"):ti,ab,kw OR ("preference"):ti,ab,kw OR (value*):ti,ab,kw OR (choice):ti,ab,kw |
| #9 | ("health state"):ti,ab,kw OR ("health status"):ti,ab,kw OR ("health status questionaire"):ti,ab,kw OR ("perception"):ti,ab,kw OR ("view-point"):ti,ab,kw |
| #10 | ("knowledge"):ti,ab,kw OR ("perspective"):ti,ab,kw |
| #11 | MeSH descriptor: [Patient Preference] explode all trees |
| #12 | MeSH descriptor: [Health Status] explode all trees |
| #13 | MeSH descriptor: [Perception] explode all trees |
| #14 | MeSH descriptor: [Knowledge] explode all trees |
| #15 | MeSH descriptor: [Choice Behavior] explode all trees |
| #16 | MeSH descriptor: [Attitude] explode all trees |
| #17 | #8 OR #9 OR #10 OR #11 OR #12 OR #13 OR #14 OR #15 OR #16 |
| #18 | ("utility"):ti,ab,kw OR ("utilities"):ti,ab,kw OR ("gambler"):ti,ab,kw OR ("visual analog scale"):ti,ab,kw OR ("elicitation"):ti,ab,kw |
| #19 | ("standard gamble"):ti,ab,kw OR ("trade off"):ti,ab,kw OR ("VAS score"):ti,ab,kw OR ("scoreboard"):ti,ab,kw OR ("scorecard"):ti,ab,kw |
| #20 | ("feeling thermometer"):ti,ab,kw |
| #21 | MeSH descriptor: [Decision Support Techniques] explode all trees |
| #22 | #18 OR #19 OR #20 OR #21 |
| #23 | ("EQ-5D"):ti,ab,kw OR ("EQ5D"):ti,ab,kw OR ("QOL"):ti,ab,kw OR ("quality adjusted life expectancy"):ti,ab,kw OR ("SF(6)"):ti,ab,kw |
| #24 | ("SF-6D"):ti,ab,kw OR ("SF-36"):ti,ab,kw OR ("attribute"):ti,ab,kw OR ("index value"):ti,ab,kw |
| #25 | ("decision support technic"):ti,ab,kw OR ("decision analyses"):ti,ab,kw |
| #26 | MeSH descriptor: [Quality of Life] explode all trees |
| #27 | MeSH descriptor: [Patient Reported Outcome Measures] explode all trees |
| #28 | MeSH descriptor: [Decision Making, Computer-Assisted] explode all trees |
| #29 | MeSH descriptor: [Decision Support Techniques] explode all trees |
| #30 | #23 OR #24 OR #25 OR #26 OR #27 OR #28 OR #29 |
| #31 | MeSH descriptor: [Decision Support Techniques] explode all trees |
| #32 | MeSH descriptor: [Decision Making] explode all trees |
| #33 | ("decision"):ti,ab,kw OR ("decision analysis"):ti,ab,kw OR ("decision maker"):ti,ab,kw OR ("decision making"):ti,ab,kw OR ("decision theories"):ti,ab,kw |
| #34 | #31 OR #32 OR #33 |
| #35 | ("questionnaire"):ti,ab,kw OR ("interview"):ti,ab,kw OR ("survey question"):ti,ab,kw OR ("focus group"):ti,ab,kw OR ("focus group discussion"):ti,ab,kw |
| #36 | MeSH descriptor: [Surveys and Questionnaires] explode all trees |
| #37 | MeSH descriptor: [Interview] in all MeSH products |
| #38 | #35 OR #36 OR #37 |
| #39 | #17 OR #22 OR #30 OR #34 OR #38 |
| #40 | #7 AND #39 |
