# Supplementary material for: Women’s values and preferences on low-molecular-weight heparin and pregnancy: a mixed-methods systematic review
Source: BMC Pregnancy Childbirth. 2022 Oct 5;22:747. doi: 10.1186/s12884-022-05042-x (PMC9533610; doi:10.1186/s12884-022-05042-x)
Supplement: Supplementary file 2 — Additional file 2. [file 12884_2022_5042_MOESM2_ESM.docx]

| **Table S1. Synthesis of quantitative outcomes** | | | | | | | | | |
| --- | --- | --- | --- | --- | --- | --- | --- | --- | --- |
| 1. Utility values | | | | | | | | | Certainty of the evidence (GRADE) |
| Preference | Instrument | No. of participants [reference] | Mean utility value (SD) [IC95] |  | | | | | 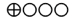  Very low certainty due to moderate RoB, indirectness and imprecision |
| **Health**  **State: Pregnancy with LMWH prophylaxis** | Measured with  visual analogue scale^1^ | 123  [(Bates 2015; Eckman 2015)] | 81(15) [78.32-83.68] |  |  |  |  |  |  |
| 2. Non-utility Values | | | | | | | | | Certainty of the evidence (GRADE) |
| Preference |  | | | | | | | |  |
| **Willing to take LMWH** | Instrument | No. of participants [reference] | % of women that would take LMWH in their Real-life scenario | % of women that would take LMWH giving a hypothetical baseline risk of recurrence = 4%) | % of women that would take LMWH giving a hypothetical baseline risk of recurrence = 10% | % of women that would take LMWH giving a hypothetical baseline risk of recurrence = 16% |  | | 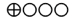  Very low certainty due to moderate RoB, indirectness and imprecision |
|  | DCE^3^ | 123  [Bates 2015; Eckman 2015] | 78.86 | 63.525 | 76.33 | 86.315 |  |  |  |
|  | Instrument | No. of participants [reference] | Median (%) of risk reduction [IQR] of LMWH: Given a fixed 16% risk of VTE |  | | | | | 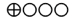  Very low certainty due to serious RoB, indirectness, inconsistency and imprecision |
|  | PTOf^4^ | 123  [Bates 2015] | 3 [1 to 6] |  |  |  |  |  |  |
|  | Instrument | No. of participants [reference] | % of women that are willing to use thromboprophylaxis for future pregnancy |  | | | | | 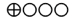  Very low certainty due to serious RoB, indirectness, inconsistency and imprecision |
|  | Researchers self-developed questionnaire | 111  [Hordern 2015] | 94,5 |  |  |  |  |  |  |
| **Beliefs towards the harms-overuse-necessity – concerns of taking LMWH** | Instrument | No. of participants [reference] | Mean (SD)- Total Harm | Mean (SD)- Total overuse | Mean (SD)- Total necessity | Mean (SD)- Total concerns | Mean (SD): Necessity- concerns differential |  | 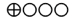  Very low certainty due to serious RoB, indirectness, inconsistency and imprecision |
|  | Belief towards medication questionnaire^2^ | 67  [Guimicheva2019] | 7.5 (2.4) | 9.8 (3.6) | 11.5 (2.9) | 10.3 (3.1) | 1.18 |  |  |
|  |  | 95  [Patel2012] | 8.22 (2.63) | 10.0 (2.90) | 14.16 (3.61) | 11.96 (3.66) | 2.20 |  |  |
| **Reason for not being adherent when using LMWH** | Instrument | No. of participants [reference] | Bruising or wound complications (% of women) | Forgetting (% of women) | Fear or dislike of needles (% of women) | feeling that the injections were not helping | stopped on medical or midwifery advice | emotional reasons for stopping, |  |
|  | Researchers self-developed questionnaire | 12  [Hordern 2015] | 25 | 16.6 | 16.6 | 16.6 | 25 | 8.3 | 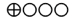  Very low certainty due to serious RoB, indirectness, inconsistency and imprecision |
| **Preference for route of administration** | Instrument | No. of participants [reference] | Preferred injecting heparin through the Teflon catheter over standard subcutaneous injections (% of women) | Preferred injecting heparin through the Teflon catheter as it caused less pain and less bruising than they experienced with twice daily injections(% of women) |  | | | |  |
|  | Researchers self-developed questionnaire | 12  [Anderson 1993] | 83.3 | 91.7 |  |  |  |  | 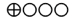  Very low certainty due to serious RoB, indirectness, inconsistency and imprecision |
| **Preferred amount of information regarding LMWH** | Instrument | No. of participants [reference] | Whether the woman had received enough information regarding LMWH (% of women (n=111)) | Would have liked more information or training before leaving hospital. (% of women (n=12)) |  |  |  |  |  |
|  | Researchers self-developed questionnaire | 111  [Hordern 2015] | 83.8 | 16.6 |  |  |  |  | 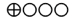  Very low certainty due to serious RoB, indirectness, inconsistency and imprecision |
| ^1^ VAS represent the value people place on different health states on an interval scale, with zero reflecting states of health equivalent to death/worst imaginable health and 100 reflecting perfect health/ best imaginable health.  ^2^ BMQ: Subscales for general harm and overuse (based on 4 items each) could have a minimum score of 4 and a maximum score of 20. Scales for specific necessity and concerns could have a minimum score of 5 and a maximum score of 25  ^3^ DCE:Participants’ willingness to receive LMWH prophylaxis through direct choice exercises using decision boards:  ^4^ PTO: Probability trade-off exercises to determine participant thresholds for accepting LMWH prophylaxis | | | | | | | | | |

| **Table S2. Synthesis of qualitative outcomes** | | | | |
| --- | --- | --- | --- | --- |
| Theme | No. of participants [reference] | | | Certainty of the evidence (GRADE-Cerqual) |
|  | 10 [Skeith 2021] | 9 [Martens 2007] | 30 [Patel 2012] |  |
|  | Finding [*quote(s)*] | Finding [*quote(s)*] | Finding [*quote(s)*] |  |
| Attitude towards the decision -making of using LMWH | *LMWH injections as “low risk” with “minimal side effects, and that, compared to the “emotional pain with loss, a little bit of physical pain from a needle is small potatoes*]  [*…It would have to be a pretty large risk for me not to do it. But if a drug or an injection increased my chances of carrying a baby full term, I can’t think of anything that I wouldn’t be at least open to talking about and finding out all about information about it first before I made a decision*]  [*I never allowed myself to plan for him, throughout my pregnancy I don’t think I ever talked about when he gets here, you know how most women are planning the nursery and everything is based on that plan and having that baby. And I never went there with him, I never allowed myself to go there, ever*]  [*We were upset, and I can’t remember which doctor I was talking to, they said it may help, it may not, so don’t … he wasn’t saying don’t get excited, but it was like this is something we’re going to try and we’ll just see what happens. So, we were kind of prepared for either way. … But it did give me the hope to think maybe this would solve the problem. So, it was a bit of excitement plus a bit of nervousness, just because you don’t know what to expect*] | [*I was happy they found a solution to why I was having the miscarriages ... I’m not really thinking of my health constantly, I’m thinking about my pregnancy. Maybe once I give birth I’ll be thinking more about myself, you know, how it could affect me. Right now, I’m not thinking about that*]  [*When the [LMWH] was started I was much more calm. I was very overwhelmed, after [LMWH] it was ok, something to help*]  [*When you want to have a baby … nothing will stop you*] | [*I’m happy to take enoxaparin during pregnancy as a preventative measure. I don’t like doing the injections but it’s manageable, and with my history it’s something I’m prepared to do to ensure a safe arrival of my baby*]  [*I am very thankful for enoxaparin and grateful it is available. It is not a problem for me to take if it means I get my babies with me after so many lost babies*]  [*As far as I am aware, enoxaparin is to ensure I remain well during pregnancy and do not have a DVT. Therefore, it is paramount that my health is taken care of and reviewed. I feel more secure and less worried about my health now I am on this medication. I find the injections uncomfortable but a small price to ensure I have a healthy pregnancy*]  [*I don’t have an issue with taking medication during pregnancy, if the unborn baby’s health is not compromised. I believe I am on enoxaparin to prevent blood clots from forming (not that they definitely would appear, but to reduce the risk of them appearing)*]  [*Definitely gives me piece of mind during pregnancy; without it I would feel very nervous about developing another DVT*] | 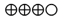  Moderate |
| Experience of using LMWH during pregnancy | [*Although it wasn’t fun injecting myself it was part of the ritual. …It felt like I was doing something instead of just waiting there to see if I would miscarry. …It felt like I at least had a very, very, very small hand in helping*]  [*I did get my pregnancy photos done, but literally a week before he was born and only because my friend said, we’ll just photoshop the bruises. We actually did take pictures with the bruises, obviously, and left them as is, because that’s part of the experience I had during the pregnancy. It’s a terrible sight, but it is what it is*] | [*But it’s horrible on the legs, I didn’t think it was going to be like that, it starts off with a little bruise and then it just gets bigger and bigger*]  [*Even though I did the treatment I was still worried. I was going to ultrasounds almost every week, I was so worried; was it really working, how is my baby?*]  [*It was worth every second*] | [*Taking enoxaparin worries me...because it’s painful]*  […*I worry about bleeding after birth and how the Clexane affects this…]*  *If there was a way to get injections for enoxaparin via an epipen type device it would be much more tolerable]* | 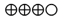  Moderate |
| Concerns about medication | [*I wanted a baby so bad, I was like I don’t care, I’ll do it… the chance of it harming me, I think my husband was a little more concerned, like it was going to do me harm, then he was like no, we’re not doing that. But we were pretty desperate at the time*]  [*I wanted another baby really badly but not enough to harm my health to take care of my first child. So, I think that would have been my no go point. If it had been something that would have permanently altered my health or my longevity, I probably wouldn’t have risked that just to have another baby*] | [*The timing is very pressuring, he told me that an hour [before or after] it doesn’t make a difference, but it is a big pressure for me to remember every single day at the same time … and what happens if I forget one day … the pressure that I shouldn’t forget, this is the most hard thing*]  [*So then you’ re worried because labor isn’ t always planned, right, and what if … I mean what if I went into labor at 33 [weeks] and I took [LMWH] yesterday or I took it today and I would worry that I would go into labor; what would ultimately happen, being on the medication and going into labor]* | [*I* *sometimes worry that in the future they find that enoxaparin has a big side-effect for my baby, for example harmful for the heart or skin*]  [*I* *do worry about the options for pain relief as have been told I need an epidural due to my hypertension too]*  [*The problem I have is that I get severe pain for 30 min after the jab, and need to lie down. Early on, I decided I would cope better at night. My husband has to administer the injection as I am terrified of needles. I wish there was another way, other than injection of taking this drug! As I am having an elective C-section, I worry that my blood may cause problems during the operation and that if I stop the Clexane before the C-section, this will cause a problem for our baby. I have suffered with severe bruising around every needle mark, this also causes me concern*] | 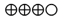  Moderate |
| Information needs to inform the decision | No data | [*I still wonder if I wouldn’t have asked for those tests, if they would have been offered to me … He [the doctor] told me, no, it’s nature that´ s all and I go home and I continue to do this [miscarry] … I wasn’t satisfied with that*]  [*No, I don ’ t remember being given any information and you are insecure about the whole subject, so you are not prepared to ask questions because you don ’ t know what to ask*]  [*She said “they [doctors] would not treat me with [LMWH]” and at 29 weeks she had a placental abruption. The kid was born much too early, they could not hold on, and the kid has problems … she tells me, “no, push them to give you [LMWH] because you don’ t want an experience like mine.*] | [*I have no issues injecting if it is safeguarding mine and the babies health but I lack some faith in the safety/side-effects/ general effects of the medicine. Published information on Clexane seems to be contradictory*] | 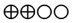  Low |
| Patient involvement in the decision-making | [*You place your faith in that doctor…that’s what makes you make that decision to go for it or not [to use LMWH injections], it depends on the kind of information you’re being given*]  [*I felt pretty involved. I didn’t feel like pressured into taking [LMWH] if I did get pregnant. It was really up to me to say I want to take the injections or not… I felt involved in the decision*] | [*She [the obstetrician] told me I had a choice of going through it or not going through it, I risk whatever reaction I have to the [LWMH] to have a good pregnancy. It’s up to me. The [LWMH] might help the pregnancy or it might not, we don’t know*] | *[I do not have a problem doing injections and was aware of the possibility of the injections before becoming pregnant. However, I think other women might benefit from more time and support around the use of Clexane in their pregnancy]* | 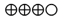  Moderate |
