# Supplementary material for: Women’s values and preferences on low-molecular-weight heparin and pregnancy: a mixed-methods systematic review
Source: BMC Pregnancy Childbirth. 2022 Oct 5;22:747. doi: 10.1186/s12884-022-05042-x (PMC9533610; doi:10.1186/s12884-022-05042-x)
Supplement: Supplementary file 3 — Additional file 3. [file 12884_2022_5042_MOESM3_ESM.docx]

Supplementary Material 2

| Table S1. Risk of Bias (GRADE instrument) | | | | | | | | | | | | | | | |
| --- | --- | --- | --- | --- | --- | --- | --- | --- | --- | --- | --- | --- | --- | --- | --- |
| Study | RoB for sampling  (Was an appropriate study sample selected from the sampling  frame?) | Comment | RoB for Attrition  (Was the attrition sufficiently low to minimize the risk of bias?) | Comment | Choice of instrument  (Was the instrument used for eliciting relative importance of  outcomes valid and reliable?) | Comment | Administration of the instrument (Was the instrument administered in the intended way?) | Comment | Outcome presentation (Was a valid representation of the outcome (health state)  utilized?) | Comment | Understanding of the instrument  (Did the researchers check the understanding of the  instrument?) | Comment | RoB for data analysis  (Were the results analyzed appropriately to avoid influence of  bias and confounding?) | Comment | Ovearall Quality assessment |
| Bates 2015 and Eckman 2015 | Moderate risk of bias | The study included pregnant women with a history of lower extremity DVT or PE who were considering thromboprophylaxis to prevent recurrent antepartum VTE. However, there was a higher proportion of women with a high education level. | Low risk of bias | From the 123 recruited, all completed the interview | Low risk of bias | The techniques used are probability trade off exercises, direct choice exercises and feeling thermometer | Low risk of bias | The deliberation of the three techniques was administered correctly | Low risk of bias | The outcomes for each of the three techniques was represented accordingly to the method. Health states were used to represent the outcomes | Low risk of bias | After presenting the descriptions and recording patient responses, interviewers reviewed participant responses to the various exercises to check for consistency in participants’ choice. When interviewers identified inconsistencies, they offered participants a chance to review and change their responses, avoiding any suggestion that responses should be changed. The reasons for any apparent inconsistencies were determined and recorded. Following this consistency check, interviewers asked participants two standardized questions to evaluate their understanding of the information provided during the interview. Interviewers also provided a rating of the extent to which they believed the respondents had a clear understanding of the questions and their confidence in this assessment. | Low risk of bias | Analytical methods were correct | 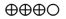  Moderate risk of bias |
| Guimicheva 2019 | Moderate risk of bias | Unclear sampling strategy. Unclear how many were approached and how many agreed to participate. | Serious risk of bias | Attrition rate >30% | Low risk of bias | The BMQ was considered  an appropriate tool to utilize for this study, as it has previously  been used in the gravid setting with enoxaparin, exploring whether  women's beliefs relate to their adherence to enoxaparin | Low risk of bias | Women were asked to return the completed questionnaire pack at  one of their follow up appointments, and were reminded to bring  completed questionnaires back by phone prior to their follow-up appointments. | Low risk of bias | The outcomes harm, overuse, necessity and concerns were presented through specific questions and valid to assess the beliefs towards the medication- heparin | Moderate risk of bias | The questionnaire was given to the women but it was not reported if researchers checked the understanding of the questionnaire; although the questionnaire is designed for self-administration | Low risk of bias | Analytical methods were correct | 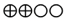  Serious risk of bias |
| Patel 2012 | Moderate risk of bias | Unclear sampling strategy. Unclear how many were approached and how many agreed to participate. Participants were recruited in a larger study “*The study was part of a larger parent study, evaluating the impact of mode of delivery and thromboprophylaxis”* | Low risk of bias | Attrition rate <10% | Low risk of bias | Validated questionnaire: BMQ was tested for these four subscales using  Cronbach s alpha, which revealed good internal consistency | Low risk of bias | The questionnaire was given to women at the time they  consented to join the study (during the antenatal period)  and women were asked to bring the completed questionnaire  to one of their subsequent hematology clinic appointments. | Low risk of bias | The outcomes harm, overuse, necessity and concerns were presented through specific questions and valid to assess the beliefs towards the medication- heparin | Moderate risk of bias | The questionnaire was given to the women but it was not reported if researchers checked the understanding of the questionnaire; although the questionnaire is designed for self-administration | Low risk of bias | Analytical methods were correct | 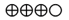  Moderate risk of bias |
| Hordern 2015 | Moderate risk of bias | Unclear sampling strategy. Unclear how many were approached and how many agreed to participate. | Serious risk of bias | Only completed surveys were used in the analysis; hence we can’t tell if those that didn’t answer had systematic differences to those that did complete the survey. | Moderate risk of bias | They don't report the consistency of the instrument as it is a questionnaire developed by researchers; they don't mention other potential studies assessing the same which could serve as useful source of validity | Low risk of bias | Yes, it was a structured interview delivered accordingly to the method | Moderate risk of bias | There was not a presentation of outcome, rather complications and discomfort of using heparin were elicited by patients in an organic way. | Low risk of bias | As it was a synchronous structured interview with women, there is no reason to think that the researchers couldn’t have clarified any questions the women had. | Low risk of bias | Analytical methods were correct | 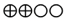  Serious risk of bias |
| Anderson, 1993 | Moderate risk of bias | Unclear sampling strategy. Unclear how many were approached and how many agreed to participate. | Serious risk of bias | Attrition rate <20% | Moderate risk of bias | Patients completed a questionnaire designed to determine which method of heparin administration they preferred and which method caused the most severe side effects. They don't report the consistency of the instrument as it is a questionnaire developed by researcher; they don't mention other potential studies assessing the same which could serve as useful source of validity | Low risk of bias | Because of the nature of this study, it was not possible to use a double-blind design. To avoid influencing the patient's questionnaire responses, physicians and nurses directly involved in the care of these patients were specifically instructed to present the study in an unbiased fashion and avoid expressing an opinion about whether they thought one of the routes of heparin administration was superior to the other. | Low risk of bias | Preference for administration route was presented | Low risk of bias | The questionnaire was given face to face to the patient and although they don't report any procedure to check understanding, there is no reason to think that the researchers couldn’t have clarified any questions the women had. | Low risk of bias | Analytical methods were correct | 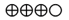  Moderate risk of bias |

| Table S2. Certainty of evidence in the importance of values and preferences | | | |
| --- | --- | --- | --- |
| Domain | Signaling questions | Outcomes | |
|  |  | Utilities (Direct techniques): Health State “Pregnancy with LMWH prophylaxis” in VAS scale (0 to 100) Note: Minimal important difference of the relative importance of outcomes such as 0.05 to 0.07 on a 0 to 1 visual analogue scale for making such judgements. Informed by one study with a population:  **N = 123 (Bates 2015 and Eckman 2015)** | |
|  |  | VAS scale: 81(15) [78,32-83,68] | Note |
| Overall Risk of Bias | Table 1. Supplementary material | Moderate risk of bias |  |
| 1.Indirectness due to PICO elements | Was the population studied matching the population of interest | Very serious | Half of the sample were neither pregnant nor planning. |
|  | Were the outcomes matching the outcomes of interest | Not serious |  |
|  | Were the options studied matching the alternative options of interest | Not serious |  |
| 2.Indirectness due to methodological elements | Were the participants answering questions directly valuing the relative importance of outcomes?  - Were direct methodologies for outcomes utilities used rather than indirect methodologies?  -Was the utility directly estimated from an instrument to elicit rather than mapped from an instrument whose purpose was not eliciting utility? | Not serious |  |
| Overall Indirectness |  | Serious |  |
| Inconsistency | | | |
| Assessment of four items  1. Similarity in point estimates  2. Overlap in confidence intervals  3. P value of the heterogeneity test (if any)  4. I (2) of the metanalysis (if any) | Are the results consistent across included studies? | Not applicable |  |
| Explore the source of inconsistency  - Consistent PICO (population, compared treatment options, outcomes)?  -Consistent methodology (study design, measurement methodologies, description of disease severity and outcomes)? | Source of Inconsistency explained | Not applicable |  |
| Subgroup estimates credibility assessment  1. Is the subgroup variable a characteristic specified at baseline?  2. Is the subgroup difference suggested by comparisons within rather than between studies?  3. Does statistical analysis suggest that chance is an unlikely explanation for the subgroup difference?  4. Did the hypothesis precede rather than follow the analysis, and include a hypothesized direction that was subsequently confirmed?  5. Was the subgroup hypothesis one of a small number tested?  6. Is the subgroup difference consistent across studies and across important outcomes?  7. Does external evidence (biological or sociological rationale) support the hypothesized subgroup difference? | Source of Inconsistency explained | Not applicable |  |
| Overall Inconsistency |  | Not applicable |  |
| Imprecision | | | |
| Inspection of sample size | Is the sample size large enough to meet the review information size? | Serious | Considering it’s a continuous variable we downgraded for small sample size. GRADE guidelines recommend above 800 participants; although, due to the comprehensive process involved with collecting values and preferences, we considered an adequate sample size to be 200 participants. |
| Inspection of confidence interval | Is the confidence interval narrow enough? |  |  |
| Overall Imprecision |  | Serious |  |
| OVERALL CERTAINTY OF THE EVIDENCE |  | 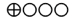  Very low certainty due to moderate RoB, indirectness and imprecision | |

| *Continue* Table S2. Certainty of evidence in the importance of values and preferences | | | |
| --- | --- | --- | --- |
| Domain | Signaling questions | Outcomes | |
|  |  | Non-utility measure: Beliefs towards medication [Mean Necessity-Concerns Differential] Informed by 2 studies with a total population of **N= 158 (n= 67[Guimicheva 2019] + n= 95 [Patel 2012])** | |
|  |  | Necessity-Concerns Differential mean= 1.18 and 2.20 | Note |
| Risk of Bias | Table 1. Supplementary material | Serious risk of bias |  |
| 1.Indirectness due to PICO elements | Was the population studied matching the population of interest | Not serious |  |
|  | Were the outcomes matching the outcomes of interest | Not serious |  |
|  | Were the options studied matching the alternative options of interest | Not serious |  |
| 2.Indirectness due to methodological elements | Were the participants answering questions directly valuing the relative importance of outcomes?  - Were direct methodologies for outcomes utilities used rather than indirect methodologies?  -Was the utility directly estimated from an instrument to elicit rather than mapped from an instrument whose purpose was not eliciting utility? | Serious | The instrument purpose was not to elicit utility |
| Overall Indirectness | | Serious |  |
| Assessment of four items  1. Similarity in point estimates  2. Overlap in confidence intervals  3. P value of the heterogeneity test (if any)  4. I (2) of the metanalysis (if any) | Are the results consistent across included studies? | Not serious |  |
| Explore the source of inconsistency  - Consistent PICO (population, compared treatment options, outcomes)?  -Consistent methodology (study design, measurement methodologies, description of disease severity and outcomes)? | Source of Inconsistency explained | Serious | There could be systematic differences due to one study including a population of both peripartum and postpartum coagulation while the other only included postpartum coagulation |
| Subgroup estimates credibility assessment  1. Is the subgroup variable a characteristic specified at baseline?  2. Is the subgroup difference suggested by comparisons within rather than between studies?  3. Does statistical analysis suggest that chance is an unlikely explanation for the subgroup difference?  4. Did the hypothesis precede rather than follow the analysis, and include a hypothesized direction that was subsequently confirmed?  5. Was the subgroup hypothesis one of a small number tested?  6. Is the subgroup difference consistent across studies and across important outcomes?  7. Does external evidence (biological o sociological rationale) support the hypothesized subgroup difference? | Source of Inconsistency explained | Not serious |  |
| Overall Inconsistency | | Serious |  |
| Inspection of sample size | Is the sample size large enough to meet the review information size? | Serious | Considering it’s a continuous variable we downgraded for small sample size. GRADE guidelines recommend above 800 participants; although, due to the comprehensive process involved with collecting values and preferences, we considered an adequate sample size to be 200 participants. |
| Inspection of confidence interval | Is the confidence interval narrow enough? |  |  |
| Overall Imprecision | | Serious | |
| OVERALL CERTAINTY OF THE EVIDENCE | | 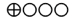  Very low certainty due to serious RoB, indirectness, inconsistency and imprecision | |

| *Continue* Table S2. Certainty of evidence in the importance of values and preferences | | | | | | | | | |
| --- | --- | --- | --- | --- | --- | --- | --- | --- | --- |
| Domain | Signaling questions | Outcomes | | | | | | | |
|  |  | Non-utility measure: Willingness to use LMWH direct choice. Informed by one study with a population: **N = 123 (Bates 2015 and BATES2015)** | | | | | | | |
|  |  | Hypothetical scenario (low; baseline risk of recurrence = 4%). % of women willing to take LMWH = 63,525 | Note | Hypothetical scenario (medium; baseline risk of recurrence = 10%). % of women willing to take LMWH = 76,33. | Note | Hypothetical scenario (high; baseline risk of recurrence = 16%. % of women willing to take LMWH = 86,315 | Note | Real-life scenario. % of women willing to take LMWH =73,18 | Note |
| Risk of Bias | Table 1. Supplementary material | Moderate risk of bias |  | Moderate risk of bias |  | Moderate risk of bias |  | Moderate risk of bias |  |
| Indirectness | | | | | | | | | |
| 1.Indirectness due to PICO elements | Was the population studied matching the population of interest | Not serious |  | Not serious |  | Not serious |  | Not serious |  |
|  | Were the outcomes matching the outcomes of interest | Not serious |  | Not serious |  | Not serious |  | Not serious |  |
|  | Were the options studied matching the alternative options of interest | Not serious |  | Not serious |  | Not serious |  | Not serious |  |
| 2.Indirectness due to methodological elements | Were the participants answering questions directly valuing the relative importance of outcomes?  - Were direct methodologies for outcomes utilities used rather than indirect methodologies?  -Was the utility directly estimated from an instrument to elicit rather than mapped from an instrument whose purpose was not eliciting utility? | Serious | The instrument purpose was not to elicit utility | Serious | The instrument purpose was not to elicit utility | Serious | The instrument purpose was not to elicit utility | Serious | The instrument purpose was not to elicit utility |
| Overall Indirectness | | Serious |  | Serious |  | Serious |  | Serious |  |
| Inconsistency | | | | | | | | | |
| Assessment of four items  1. Similarity in point estimates  2. Overlap in confidence intervals  3. P value of the heterogeneity test (if any)  4. I (2) of the metanalysis (if any) | Are the results consistent across included studies? | Not applicable |  | Not applicable |  | Not applicable |  | Not applicable |  |
| Explore the source of inconsistency  - Consistent PICO (population, compared treatment options, outcomes)?  -Consistent methodology (study design, measurement methodologies, description of disease severity and outcomes)? | Source of Inconsistency explained | Not applicable |  | Not applicable |  | Not applicable |  | Not applicable |  |
| Subgroup estimates credibility assessment  1. Is the subgroup variable a characteristic specified at baseline?  2. Is the subgroup difference suggested by comparisons within rather than between studies?  3. Does statistical analysis suggest that chance is an unlikely explanation for the subgroup difference?  4. Did the hypothesis precede rather than follow the analysis, and include a hypothesized direction that was subsequently confirmed?  5. Was the subgroup hypothesis one of a small number tested?  6. Is the subgroup difference consistent across studies and across important outcomes?  7. Does external evidence (biological o sociological rationale) support the hypothesized subgroup difference? | Source of Inconsistency explained | Not applicable |  | Not applicable |  | Not applicable |  | Not applicable |  |
| Overall Inconsistency |  | Not applicable |  | Not applicable |  | Not applicable |  | Not applicable |  |
| Imprecision | | | | | | | | | |
| Inspection of sample size | Is the sample size large enough to meet the review information size? | Serious | Considering it’s a continuous variable we downgraded for small sample size. GRADE guidelines recommend above 800 participants; although, due to the comprehensive process involved with collecting values and preferences, we considered an adequate sample size to be 200 participants. | Serious | Considering it’s a continuous variable we downgraded for small sample size. GRADE guidelines recommend above 800 participants; although, due to the comprehensive process involved with collecting values and preferences, we considered an adequate sample size to be 200 participants. | Serious | Considering it’s a continuous variable we downgraded for small sample size. GRADE guidelines recommend above 800 participants; although, due to the comprehensive process involved with collecting values and preferences, we considered an adequate sample size to be 200 participants. | Serious | Considering it’s a continuous variable we downgraded for small sample size. GRADE guidelines recommend above 800 participants; although, due to the comprehensive process involved with collecting values and preferences, we considered an adequate sample size to be 200 participants. |
| Inspection of confidence interval | Is the confidence interval narrow enough? |  |  |  |  |  |  |  |  |
| Overall Imprecision |  | Serious |  | Serious |  | Serious |  | Serious |  |
| OVERALL CERTAINTY OF THE EVIDENCE |  | 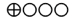  Very low certainty due to moderate RoB, indirectness and imprecision | | | | | | | |

| *Continue* Table S2. Certainty of evidence in the importance of values and preferences | | | |
| --- | --- | --- | --- |
| Domain | Signaling questions | Outcomes | |
|  |  | Non-utilities: Threshold reduction in VTE risk at which women were willing to accept use of LMWH. Informed by one study with a population **N = 123 (BATES2015 and Eckman 2015)** | |
|  |  | Given a fixed 16% risk Median (%) of risk reduction [IQR] = 3 [1 to 6] | Note |
| Risk of Bias | Table 1. Supplementary material | Moderate risk of bias |  |
| 1.Indirectness due to PICO elements | Was the population studied matching the population of interest | Not serious |  |
|  | Were the outcomes matching the outcomes of interest | Not serious |  |
|  | Were the options studied matching the alternative options of interest | Not serious |  |
| 2.Indirectness due to methodological elements | Were the participants answering questions directly valuing the relative importance of outcomes?  - Were direct methodologies for outcomes utilities used rather than indirect methodologies?  -Was the utility directly estimated from an instrument to elicit rather than mapped from an instrument whose purpose was not eliciting utility? | Serious | The instrument purpose was not to elicit utility |
| Overall Indirectness | | Serious |  |
| Inconsistency |  |  |  |
| Assessment of four items  1. Similarity in point estimates  2. Overlap in confidence intervals  3. P value of the heterogeneity test (if any)  4. I (2) of the metanalysis (if any) | Are the results consistent across included studies? | Not applicable |  |
| Explore the source of inconsistency  - Consistent PICO (population, compared treatment options, outcomes)?  -Consistent methodology (study design, measurement methodologies, description of disease severity and outcomes)? | Source of Inconsistency explained | Not applicable |  |
| Subgroup estimates credibility assessment  1. Is the subgroup variable a characteristic specified at baseline?  2. Is the subgroup difference suggested by comparisons within rather than between studies?  3. Does statistical analysis suggest that chance is an unlikely explanation for the subgroup difference?  4. Did the hypothesis precede rather than follow the analysis, and include a hypothesized direction that was subsequently confirmed?  5. Was the subgroup hypothesis one of a small number tested?  6. Is the subgroup difference consistent across studies and across important outcomes?  7. Does external evidence (biological o sociological rationale) support the hypothesized subgroup difference? | Source of Inconsistency explained | Not applicable |  |
| Overall Inconsistency |  | Not applicable |  |
| Inspection of sample size | Is the sample size large enough to meet the review information size? | Serious | Considering it’s a continuous variable we downgraded for small sample size. GRADE guidelines recommend above 800 participants; although, due to the comprehensive process involved with collecting values and preferences, we considered an adequate sample size to be 200 participants. |
| Inspection of confidence interval | Is the confidence interval narrow enough? |  |  |
| Overall Imprecision |  | Serious |  |
| OVERALL CERTAINTY OF THE EVIDENCE |  | 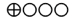  Very low certainty due to moderate RoB, indirectness and imprecision | |

| *Continue* Table2. Certainty of evidence in the importance of values and preferences | | | | | | | | | |
| --- | --- | --- | --- | --- | --- | --- | --- | --- | --- |
| Domain | Signaling questions | Outcomes | | | | | | | |
|  |  | Non-utilities- Questionnaires | | | | | | | |
|  |  | Willingness to use thromboprophylaxis for future pregnancy (% dichotomous variable)  **N= 111**  **Hordern 2015** | Note | Discomfort or complication of using LMWH (%, continous variable)  **N= 12**  **Hordern 2015** | Note | Adequacy of the information regarding LMWH (% dichotomous variable)  **N= 12**  **Hordern 2015** | Note | Preference for route of administration (% dichotomous variable)  **N= 12**  **Anderson 1993** | Note |
| Risk of Bias | Table 1. Supplementary material | Serious risk of bias |  | Serious risk of bias |  | Serious risk of bias |  | Moderate risk of bias |  |
| Indirectness |  |  |  |  |  |  |  |  |  |
| 1.Indirectness due to PICO elements | Was the population studied matching the population of interest | Not serious |  | Not serious |  | Not serious |  | Not serious |  |
|  | Were the outcomes matching the outcomes of interest | Not serious |  | Not serious |  | Not serious |  | Not serious |  |
|  | Were the options studied matching the alternative options of interest | Not serious |  | Not serious |  | Not serious |  | Not serious |  |
| 2.Indirectness due to methodological elements | Were the participants answering questions directly valuing the relative importance of outcomes?  - Were direct methodologies for outcomes utilities used rather than indirect methodologies?  -Was the utility directly estimated from an instrument to elicit rather than mapped from an instrument whose purpose was not eliciting utility? | Serious | The instrument purpose was not to elicit utility | Serious | The instrument purpose was not to elicit utility | Serious | The instrument purpose was not to elicit utility | Serious | The instrument purpose was not to elicit utility |
| Overall Indirectness | | Serious |  | Serious |  | Serious |  | Serious |  |
| Inconsistency |  |  |  |  |  |  |  |  |  |
| Assessment of four items  1. Similarity in point estimates  2. Overlap in confidence intervals  3. P value of the heterogeneity test (if any)  4. I (2) of the metanalysis (if any) | Are the results consistent across included studies? | Not applicable |  | Not applicable |  | Not applicable |  | Not applicable |  |
| Explore the source of inconsistency  - Consistent PICO (population, compared treatment options, outcomes)?  -Consistent methodology (study design, measurement methodologies, description of disease severity and outcomes)? | Source of Inconsistency explained | Not applicable |  | Not applicable |  | Not applicable |  | Not applicable |  |
| Subgroup estimates credibility assessment  1. Is the subgroup variable a characteristic specified at baseline?  2. Is the subgroup difference suggested by comparisons within rather than between studies?  3. Does statistical analysis suggest that chance is an unlikely explanation for the subgroup difference?  4. Did the hypothesis precede rather than follow the analysis, and include a hypothesized direction that was subsequently confirmed?  5. Was the subgroup hypothesis one of a small number tested?  6. Is the subgroup difference consistent across studies and across important outcomes?  7. Does external evidence (biological o sociological rationale) support the hypothesized subgroup difference? | Source of Inconsistency explained | Not applicable |  | Not applicable |  | Not applicable |  | Not applicable |  |
| Overall Inconsistency |  | Not applicable |  |  |  | Not applicable |  | Not applicable |  |
| Imprecision |  |  |  |  |  |  |  |  |  |
| Inspection of sample size | Is the sample size large enough to meet the review information size? | Serious | Considering it’s a continuous variable we downgraded for small sample size. GRADE guidelines recommend above 800 participants; although, due to the comprehensive process involved with collecting values and preferences, we considered an adequate sample size to be 200 participants. | Very serious | Considering it’s a continuous variable we downgraded for small sample size. GRADE guidelines recommend above 800 participants; although, due to the comprehensive process involved with collecting values and preferences, we considered an adequate sample size to be 200 participants. | Very serious | Considering it’s a continuous variable we downgraded for small sample size. GRADE guidelines recommend above 800 participants; although, due to the comprehensive process involved with collecting values and preferences, we considered an adequate sample size to be 200 participants. | Very serious | Considering it’s a continuous variable we downgraded for small sample size. GRADE guidelines recommend above 800 participants; although, due to the comprehensive process involved with collecting values and preferences, we considered an adequate sample size to be 200 participants. |
| Inspection of confidence interval | Is the confidence interval narrow enough? |  |  |  |  |  |  |  |  |
| Overall Imprecision |  | Serious |  | Very serious |  | Very serious |  | Very serious |  |
| OVERALL CERTAINTY OF THE EVIDENCE |  | 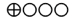  Very low certainty due to serious RoB, indirectness, inconsistency and imprecision | | | | | | | |

| Table S3: Methodological quality assessment (CASP instrument) | | | | |
| --- | --- | --- | --- | --- |
| SECTION | ITEM | CRITERIA | STUDY | |
|  |  |  | Skeith 2021 | Martens 2007 |
| Section A: Are the results valid? | 1 | Was there a clear statement of the aims of the research? • what was the goal of the research • why it was thought important • its relevance | YES | YES |
|  |  | Comments | There was a clear statement of the aims of the study: “Given the clinical equipoise, we sought to understand how patients and physicians navigate the decision-making process for use of LMWH and/or ASA in pregnancy." | There was a clear statement of the aims of the research: The purpose of this study was to better understand the experience of thrombophilia in pregnancy by considering the events leading up to diagnosis, the impact of treatment, the perceived needs, and the strategies used by pregnant women coping with thrombophilia. |
|  | 2 | Is a qualitative methodology appropriate? •If the research seeks to interpret or illuminate the actions and/or subjective experiences of research participants • Is qualitative research the right methodology for addressing the research goal | YES | YES |
|  |  | Comments | Authors conducted a qualitative study using thematic analysis informed by the key tenets of constructivist grounded theory, to better understand this socially situated research question. | The research design: phenomenological approach was appropriate to explore the experience of thrombophilia during pregnancy and using LMWH |
| Section A: Is it worth continuing? | 3 | Was the research design appropriate to address the aims of the research? • if the researcher has justified the research design (e.g. have they discussed how they decided which method to use) | YES | YES |
|  |  | Comments | In the introduction section they discuss how other methods to collect preferences (quantitative methodologies- qualitative data) were not appropriate to capture a full understanding of factors influencing decision-making. In a related study, researchers evaluated different decision-making methods for the use of LMWH prophylaxis in pregnant patients with past venous thromboembolism (VTE) and found that there was a high level of discordance between what was predicted using decision modeling and what women chose to do. Two different methods (health state utility and probability trade-off exercises) could not predict or account for the nuanced decision-making around thromboprophylaxis. Thus, our collective understanding of this decision-making process remains incomplete[...]Contextual factors about a patient’s situation provided more nuanced decision-making and helped to identify a patient-led decision-making experience. | Such studies provide a plethora of rich data, which facilitates a better understanding of the participants’ beliefs, values, struggles, and triumphs throughout the trajectory of their experience. |
|  | 4 | Was the recruitment strategy appropriate to the aims of the research? If the researcher has explained how the participants were selected • If they explained why the participants they selected were the most appropriate to provide access to the type of knowledge sought by the study • If there are any discussions around recruitment (e.g. why some people chose not to take part) | NO | NO |
|  |  | Comments | Authors include both pregnant and not pregnant women. There is no description on how much time elapsed from the last pregnancy to inclusion. It may be some sort of recall bias regarding the process of decision-making. Only 2 of the 10 included women were actually pregnant. | Authors acknowledge the limitation that from the 9 women included, 4 have had a successful pregnancy. It is possible that experience of women who experienced fetal loss while on heparin may have a different experience (selection bias). |
|  | 5 | Was the data collected in a way that addressed the research issue? • If the setting for the data collection was justified • If it is clear how data were collected (e.g. focus group, semi-structured interview etc.) • If the researcher has justified the methods chosen• If the researcher has made the methods explicit (e.g. for interview method, is there an indication of how interviews are conducted, or did they use a topic guide) • If methods were modified during the study. If so, has the researcher explained how and why • If the form of data is clear (e.g. tape recordings, video material, notes etc.) • If the researcher has discussed saturation of data (when applicable) | CAN’T TELL | CAN’T TELL |
|  |  | Comments | Not enough information is provided. Authors state that interviews were audio recorded and transcribed verbatim. They also provide the interview guide. However, there is no information about the setting where interviews were conducted nor the length of time they took. Also, there is no justification on why they conducted interviews instead of focus groups. There is also no justification on why some interviews were conducted face-to-face and some were conducted by phone. No discussion about data saturation | Setting of data collection was justified. But it lacks some information: 1) no justification of the method used (why they used semi structured interviews vs other techniques such as in-depth interviews or focus groups). 2)authors did not mention the use of a topic guide to conduct the semi structured interviews. 3) No mention of data saturation in the methods section although it seems it was reached "data collection concluded after a 4-month period at which time similar categories were emerging from the interviews..." |
|  | 6 | Has the relationship between researcher and participants been adequately considered? [Was the role of the researcher/ reflexivity described?] If the researcher critically examined their own role, potential bias and influence during (a) formulation of the research questions (b) data collection, including sample recruitment and choice of location • How the researcher responded to events during the study and whether they considered the implications of any changes in the research design | NO | NO |
|  |  | Comments | No discussion about the relationship between researcher and participants. Researchers who conducted the interviews were a thrombosis medicine researcher and an obstetrician/gynecologist. Their point of view may have influenced how the interviews were conducted and the analysis performed. | Researcher did not examine their own role, potential bias and influence. |
| Section B: What are the results? | 7 | Have ethical issues been taken into consideration? If there are sufficient details of how the research was explained to participants for the reader to assess whether ethical standards were maintained • If the researcher has discussed issues raised by the study (e.g. issues around informed consent or confidentiality or how they have handled the effects of the study on the participants during and after the study) • If approval has been sought from the ethics committee | CAN’T TELL | CAN’T TELL |
|  |  | Comments | No mention to how participants were informed. No mention of any informed consent. However, the study was approved by the OHSNREB | Despite ethical approval being obtained, in the manuscript there are no details about how the research was explained to participants and there is no mention of informed consent or confidentiality of data. |
|  | 8 | Was the data analysis sufficiently rigorous? • If there is an in-depth description of the analysis process • If thematic analysis is used. If so, is it clear how the categories/themes were derived from the data • Whether the researcher explains how the data presented were selected from the original sample to demonstrate the analysis process • If sufficient data are presented to support the findings • To what extent contradictory data are taken into account • Whether the researcher critically examined their own role, potential bias and influence during analysis and selection of data for presentation | YES | YES |
|  |  | Comments | Data was analyzed iteratively using NVivo 12 software to code the data and assist in organizing the codes as we conducted constant comparative analysis leading to more conceptual categories and the relationships between those categories (QSR International, Version 12, 2019). We generated codes through our line by line review of the transcripts independently. As part of an iterative process, we had analytic meetings that compared the similarities and differences between our generated codes, and then we collapsed codes into larger categories. We then used Mind Node software to reorganize and explore the relationships between categories before arriving to the final themes identified. | Data analysis was rigorous: Thematic analysis was used throughout the process of interviewing, transcribing, and reviewing the data. Transcripts and field notes were examined line by line and key statements regarding participants’ experiences highlighted and coded. Codes were then defined, categorized, and compiled into themes. Categories were reviewed for overlap, compared between subjects, and continually refined. Trustworthiness (Lincoln & Guba, 1985) was enhanced by audiotaping and transcribing each interview verbatim and by completing field notes within 24 hours of each interview. In addition, each step of the analysis was reviewed and validated by the coauthor. |
|  | 9 | Is there a clear statement of findings? • If the findings are explicit • If there is adequate discussion of the evidence both for and against the researcher’s arguments • If the researcher has discussed the credibility of their findings (e.g. triangulation, respondent validation, more than one analyst) • If the findings are discussed in relation to the original research question | YES | YES |
|  |  | Comments | Findings were explicit, and findings were adequately discussed both for and against researcher's arguments; data was extracted independently line by line by two researchers; and findings were discussed in relation to the original research strategy | Categories were compared between subjects. It seems that only one person conducted the analysis, but each step of the analysis was reviewed and validated by the co-author. |
| Section C: Will the results help locally? | 10 | How valuable is the research? Answer is in text. Comments [Record your reasons for your answer] • If the researcher discusses the contribution the study makes to existing knowledge or understanding (e.g. do they consider the findings in relation to current practice or policy, or relevant research-based literature • If they identify new areas where research is necessary• If the researchers have discussed whether or how the findings can be transferred to other populations or considered other ways the research may be used | YES | YES |
|  |  | Comments | The results of our study may impact future plans and the ability to conduct randomized controlled trials evaluating LMWH and ASA therapy. | Research includes a clinical implications section and a general statement: “The study findings provide insight into the lived experiences of pregnant women with thrombophilia and offer several implications for health professionals caring for this population.” |
|  | 11 | Overall assessment | Unmet Criteria 4 and 6 | Unmet Criteria 4 and 6 |
|  |  | Comments | Concerns regarding selection bias and the consideration of the relationship between researchers and participants. | Concerns regarding selection bias and the consideration of the relationship between researchers and participants. |

| Table S4: CERQual Evidence Profile | | | | | | | | | | | |
| --- | --- | --- | --- | --- | --- | --- | --- | --- | --- | --- | --- |
| Summary of Qualitative Findings (SoQF) | Studies contributing to the review finding | Methodological limitations | Coherence | Adequacy | Relevance | | | | | CERQual assessment of confidence in the evidence | Explanation of CERQual assessment |
| Themes |  |  |  |  |  |  |  |  |  |  |  |
|  |  |  |  |  | Direct relevance | Indirect relevance | Partial relevance | Unclear relevance | Overall Relavance |  |  |
| Attitude towards the decision -making of using LMWH | BATES.2021  Martens.2007  Patel 2012 | Moderate methodological limitations  2 studies with moderate (unclear recruitment and sampling) | No or very minor concerns | Minor concerns: Elements of the underlying data are well-defined presenting few demands for data richness; however, there is a demand for data quantity | Skeith.2021  Martens.2007  Patel 2012 |  |  | Patel 2012 | Moderate: This finding was informed by participants who were using LMWH to avoid miscarriage, hence women at high risk; the process of weighing pros and cons would be different for women at low risk. Regarding population characteristics, there is evidence that race is an important modifier of pregnancy loss in women with thrombophilia. | 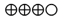  Moderate | Moderate concerns regarding methodological limitations and relevance of the data |
| Experience of using LMWH during pregnancy | Bates.2021  Martens.2007  Patel 2012 | Moderate methodological limitations  2 studies with moderate (unclear recruitment and sampling) | Moderate concerns: Key aspects of the underlying data may be vaguely defined and the alternative explanation is that women who had not previous experience with using LMWH were making the choice only based on miscarriage experience | Minor concerns: Elements of the underlying data are well-defined presenting few demands for data richness; however, there is a demand for data quantity | Skeith.2021  Martens.2007  Patel 2012 |  |  |  | No or very minor concerns | 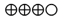  Moderate | Moderate concerns regarding methodological limitations and coherence of the data |
| Concerns about medication | Bates.2021  Martens.2007  Patel 2012 | Moderate methodological limitations  2 studies with moderate (unclear recruitment and sampling) | Moderate concerns: Key aspects of the underlying data related to safety may be vaguely defined and the alternative explanation is that women who were not first- time mother may have a higher priority of her own health vs the unborn | Minor concerns: Elements of the underlying data are well-defined presenting few demands for data richness; however, there is a demand for data quantity | Skeith.2021  Patel 2012 |  |  |  | No or very minor concerns | 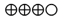  Moderate | Moderate concerns regarding methodological limitations and coherence of the data |
| Information needs to inform the decision | Martens.2007  Patel 2012 | Moderate methodological limitations  2 studies with moderate (unclear recruitment and sampling) | No or very minor concerns | Moderate concerns: Elements of the underlying data are well-defined presenting few demands for data richness; however, there is a demand for data quantity | Martens.2007  Patel 2012 |  | Patel 2012 |  | Minor concerns: Limited data coming from high income countries were education level and health literacy would affect the relevance (2) | 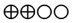  Low | Moderate concerns regarding methodological limitations and adequacy of the data. Minor concerns regarding the relevance of the data |
| Patient involvement in the decision-making | Bates.2021  Martens.2007  Patel 2012 | Moderate methodological limitations  2 studies with moderate (unclear recruitment and sampling) | No or very minor concerns | Minor concerns: Elements of the underlying data are well-defined presenting few demands for data richness; however, there is a demand for data quantity. | Skeith.2021  Martens.2007 |  |  | Patel 2012 | No or very minor concerns | 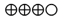  Moderate | Moderate concerns regarding methodological limitations and relevance of data |
| 1. Blondon M, Harrington LB, Righini M, Boehlen F, Bounameaux H, Smith NL. Racial and ethnic differences in the risk of postpartum venous thromboembolism: A population-based, case-control study. Journal of Thrombosis and Haemostasis. 2014 Dec 1;12(12):2002–9  2. Vahdat S, Hamzehgardeshi L, Hessam S, Hamzehgardeshi Z. Patient involvement in health care decision making: A review. Iranian Red Crescent Medical Journal. 2014;16(1).  ^a^ When describing relevance judgements, we considered the following prompts to help elucidate ‘partial’ and/or ‘unclear’ relevance: phenomenon of interest, population (including subgroups), setting, place, intervention, findings. | | | | | | | | | | | |
